# TRAF1 Coordinates Polyubiquitin Signaling to Enhance Epstein-Barr Virus LMP1-Mediated Growth and Survival Pathway Activation

**DOI:** 10.1371/journal.ppat.1004890

**Published:** 2015-05-21

**Authors:** Hannah Greenfeld, Kaoru Takasaki, Michael J. Walsh, Ina Ersing, Katharina Bernhardt, Yijie Ma, Bishi Fu, Camille W. Ashbaugh, Jackson Cabo, Sarah B. Mollo, Hufeng Zhou, Shitao Li, Benjamin E. Gewurz

**Affiliations:** 1 Division of Infectious Diseases, Department of Medicine, Brigham and Women’s Hospital, Boston, Massachusetts, United States of America; 2 Division of Immunology, Department of Microbiology and Immunobiology, Harvard Medical School, Boston, Massachusetts, United States of America; University of North Carolina at Chapel Hill, UNITED STATES

## Abstract

The Epstein-Barr virus (EBV) encoded oncoprotein Latent Membrane Protein 1 (LMP1) signals through two C-terminal tail domains to drive cell growth, survival and transformation. The LMP1 membrane-proximal TES1/CTAR1 domain recruits TRAFs to activate MAP kinase, non-canonical and canonical NF-kB pathways, and is critical for EBV-mediated B-cell transformation. TRAF1 is amongst the most highly TES1-induced target genes and is abundantly expressed in EBV-associated lymphoproliferative disorders. We found that TRAF1 expression enhanced LMP1 TES1 domain-mediated activation of the p38, JNK, ERK and canonical NF-kB pathways, but not non-canonical NF-kB pathway activity. To gain insights into how TRAF1 amplifies LMP1 TES1 MAP kinase and canonical NF-kB pathways, we performed proteomic analysis of TRAF1 complexes immuno-purified from cells uninduced or induced for LMP1 TES1 signaling. Unexpectedly, we found that LMP1 TES1 domain signaling induced an association between TRAF1 and the linear ubiquitin chain assembly complex (LUBAC), and stimulated linear (M1)-linked polyubiquitin chain attachment to TRAF1 complexes. LMP1 or TRAF1 complexes isolated from EBV-transformed lymphoblastoid B cell lines (LCLs) were highly modified by M1-linked polyubiqutin chains. The M1-ubiquitin binding proteins IKK-gamma/NEMO, A20 and ABIN1 each associate with TRAF1 in cells that express LMP1. TRAF2, but not the cIAP1 or cIAP2 ubiquitin ligases, plays a key role in LUBAC recruitment and M1-chain attachment to TRAF1 complexes, implicating the TRAF1:TRAF2 heterotrimer in LMP1 TES1-dependent LUBAC activation. Depletion of either TRAF1, or the LUBAC ubiquitin E3 ligase subunit HOIP, markedly impaired LCL growth. Likewise, LMP1 or TRAF1 complexes purified from LCLs were decorated by lysine 63 (K63)-linked polyubiqutin chains. LMP1 TES1 signaling induced K63-polyubiquitin chain attachment to TRAF1 complexes, and TRAF2 was identified as K63-Ub chain target. Co-localization of M1- and K63-linked polyubiquitin chains on LMP1 complexes may facilitate downstream canonical NF-kB pathway activation. Our results highlight LUBAC as a novel potential therapeutic target in EBV-associated lymphoproliferative disorders.

## Author Summary

The linear ubiquitin assembly complex (LUBAC) plays crucial roles in immune receptor-mediated NF-kB and MAP kinase pathway activation. Comparatively little is known about the extent to which microbial pathogens use LUBAC to activate downstream pathways. We demonstrate that TRAF1 enhances EBV oncoprotein LMP1 TES1/CTAR1 domain mediated MAP kinase and canonical NF-kB activation. LMP1 TES1 signaling induces association between TRAF1 and LUBAC, and triggers M1-polyubiquitin chain attachment to TRAF1 complexes. TRAF1 and LMP1 complexes are decorated by M1-polyubiquitin chains in LCL extracts. TRAF2 plays a key role in LMP1-induced LUBAC recruitment and M1-chain attachment to TRAF1 complexes. TRAF1 and LMP1 complexes are modified by lysine 63-linked polyubiquitin chains in LCL extracts, and TRAF2 is a target of LMP1-induced K63-ubiquitin chain attachment. Thus, the TRAF1:TRAF2 heterotrimer may coordinate ubiquitin signaling downstream of TES1. Depletion of TRAF1 or the LUBAC subunit HOIP impairs LCL growth and survival. Thus, although TRAF1 is the only TRAF without a RING finger ubiquitin ligase domain, TRAF1 nonetheless has important roles in ubiqutin-mediated signal transduction downstream of LMP1. Our work suggests that LUBAC is important for EBV-driven B-cell proliferation, and suggests that LUBAC may be a novel therapeutic target in EBV-associated lymphoproliferative disorders.

## Introduction

Epstein-Barr virus (EBV) is an oncogenic gamma-herpesvirus that is the causative agent of infectious mononucleosis. While EBV infection generally results in subclinical lifelong infection for most individuals, EBV is nonetheless associated with multiple human malignancies [1,2,3,4,5]. These include Hodgkin lymphoma, post-transplant lymphoproliferative disease (PTLD), and HIV-associated lymphomas. In these malignancies, the principal EBV oncoprotein, Latent Membrane Protein 1 (LMP1), is often expressed. LMP1 constitutively activates growth and survival pathways by mimicking CD40 signaling [6,7,8]. CD40 is a member of the tumor necrosis factor receptor (TNFR) family and serves as a key B-cell costimulatory molecule [9,10,11]. LMP1 expression transforms rodent fibroblasts and murine B-cells, and is necessary for EBV-mediated conversion of human B lymphocytes into immortalized lymphoblastoid cell lines (LCLs) [12,13,14,15,16,17].

LMP1 is comprised of a 24-residue N-terminal cytoplasmic tail, 6 transmembrane domains (TM), and a 200 residue C-terminal cytoplasmic tail. Deletion of the LMP1 N-terminus abrogates EBV-mediated B-cell transformation and alters LMP1 localization [18]. However, specific roles of the LMP1 N-terminus remain to be defined at the molecular level. The LMP1 TM domains drive assembly of LMP1 signalosome oligomers, which constitutively signal in a ligand independent manner from C-terminal tail domains [19,20,21,22,23]. The membrane proximal Transformation Effector Site (TES1)/C-terminal Activation Domain (CTAR1) spans residues 186–231. The TES1 P_204_QQAT_210_ motif binds directly to the TRAF domain of TNF receptor associated factor 2 (TRAF2), and likely also to conserved residues in the TRAFs 1, 3, and 5 domains [24]. The LMP1 PQQAT motif is necessary for TES1/CTAR1-medaited MAP kinase, canonical and non-canonical NF-kB pathway activation [24,25,26,27,28,29,30]. LMP1 TES1 activates additional pathways, including PI3K [31]. The LMP1 TES2/CTAR2 domain spans residues 351–386 and uses TRAF6 to further activate canonical NF-kB, MAP kinase, and IRF7 pathways [32,33,34]. The LMP1 CTAR3 domain, located between residues 231 and 350, associates with UBC9 and contributes to LMP1-mediated cellular migration [35]. The composition of TRAF complexes in LMP1-expressing cells has yet to be fully defined, and important components have recently been described [36,37,38,39].

LMP1 TES1 is critical for primary B lymphocyte growth transformation, since recombinant EBV that lacks LMP1 residues 185–211 does not initiate LCL outgrowth in tissue culture [25,40]. By contrast, the N-terminal 231 LMP1 residues support EBV-mediated B-cell outgrowth for up to five weeks in culture [41], and long-term on fibroblast feeder layers [40]. Interestingly, while LMP1 regulates the expression of a wide-array of host cell genes [42,43,44,45], a subset are uniquely induced by TES1 signaling. Notably, TRAF1 is amongst the earliest and most highly up-regulated LMP1 B-cell targets [29,46]. TRAF1 is abundantly expressed in EBV-infected immunoblasts in patients with infectious mononucleosis [47], and is also highly expressed in EBV-associated PTLD and Hodgkin lymphoma, where TRAF1 serves as an important biomarker [47,48,49]. TRAF1 expression is higher in EBV-positive Hodgkin lymphoma than in EBV-negative tumor samples [49]. Some nasopharyngeal carcinomas express LMP1 and TRAF1 [50]. In LCLs, most TRAF1 is associated with LMP1, either as TRAF1 homotrimers, or as TRAF1:TRAF2 heterotrimers [29]. LMP1 and TRAF1 co-localize in acquired immunodeficiency syndrome (AIDS)-associated lymphoma, PTLD, and Hodgkin lymphoma samples [51]. Despite these intriguing associations, little is known about the extent to which TRAF1 plays a pathogenic role in EBV-associated malignancy.

LMP1 TES1-mediated activation of the JNK/AP-1 axis is critically dependent on both TRAF1 and TRAF2 [52]. Although TRAF1 is the only TRAF family member that is not equipped with an N-terminal RING finger domain, TRAF1 is nonetheless the only TRAF that co-activates TES1-mediated NF-kB and JNK pathway activation [29,52]. Likewise, TRAF1 enhances signaling from the TNF receptor family member 4-1BB, and is important for CD8+ T-cell responses during chronic viral infection [53,54]. The mechanism by which TRAF1 enhances LMP1 signaling remains incompletely understood.

The role of TRAF proteins in NF-kB and MAP kinase pathway activation is perhaps best understood in the context of TNF receptor signaling. Upon TNF stimulation, TNF receptor 1 binds TRADD, which in turn recruits TRAF2, cIAP1 and cIAP2, and subsequently the linear ubiquitin assembly complex (LUBAC) [55]. LUBAC is comprised of two ring-in-between-ring E3 ligases subunits, HOIP and HOIL-1L, and the scaffold protein SHARPIN. LUBAC catalyzes a peptide bond between the N-terminal methionine alpha-amino group of one ubiquitin molecule and the C-terminal glycine of another ubiquitin molecule [56]. Linear (Met1 or M1) linked poly-Ub (pUb) chains stabilize the TNFR1 complex and enable recruitment of downstream activators [57,58]. LUBAC is also recruited to CD40 in a TRAF2-dependent manner, and SHARPIN deficiency impairs CD40-mediated NF-kB activation [55,59,60]. TNFR1 induces linear ubiquitination of RIP1 and IKK-gamma, and CD40 signaling also induces linear ubiquitination of IKK-gamma [55]. Interestingly, the IKK-gamma UBAN domain strongly associates with M1 chains, and thereby recruits the IKK-alpha and IKK-beta kinases to activated receptors to activate canonical NF-kB [61,62,63]. The extent to which M1-linked pUb chains participate in LMP1 signaling remains unknown.

To gain insight into the molecular mechanism by which TRAF1 functions downstream of LMP1, we performed proteomic analysis of immuno-purified TRAF1 complexes from cells uninduced or induced for LMP1 1–231 expression. We identified LUBAC components as high-confidence TRAF1 interactors in cells that express LMP1 residues 1–231, and found that TRAF1 and LMP1 complexes are highly M1-pUb linked in LCLs. LMP1 and TRAF1 complexes immuno-purified from LCL extracts were each K63-pUb chain modified.

## Materials and Methods

### Cell lines, plasmids, antibodies

Human embryonic kidney (HEK)-293 cells were obtained from Elliott Kieff (Brigham and Women’s Hospital, Boston, MA). 293 cells with inducible LMP1 1–231 expression were constructed, using a tightly regulated Tet-on inducible system that was previously described [43]. Briefly, the inducible system for LMP1 1–231 expression consisted of three parts: (i) an untagged LMP1 1–231 cDNA with stop codon after residue 231, cloned into the tetracycline-regulated pTRE-tight vector (Clontech); (ii) a tetracycline suppressor (tTS) that binds Tet operator sites in the absence of tetracycline and silences expression; (iii) a reverse tetracycline transactivator fused to the 4-hydroxy tamoxifen (4HT) ligand-binding domain (rTTA M2). LMP1 expression was induced by addition of doxycycline (1ug/ml) and 4HT (100 nM). A clone that had undetectable LMP1 1–231 expression at baseline, and inducible LMP 1–231 expression upon doxycycline and 4HT addition, was selected. Conditional LMP1 1–231 cell lines with stable N-terminal FLAG epitope-tagged TRAF1, TRAF2, TRAF3, or green fluorescence protein (GFP) expression were established by murine stem cell leukemia virus (MSCV) transduction and puromycin selection, as previously described [64–65]. GM12878 cells were provided by Elliott Kieff. MSCV transduction was also used to derive GM12878 LCLs with stably expressed N-terminally epitope-tagged TRAF1, GFP, SHARPIN, IKK-gamma or IKK-epsilon. LCLs that express N-terminal FLAG-tagged LMP1 at physiological levels, in place of untagged LMP1, were previously described [66], and were generously provided by Elliott Kieff. Briefly, a FLAG-tagged LMP1 cDNA was recombined into the P3HR-1 genome LMP1 locus by second site homologous recombination. 293 cell lines were cultured in DMEM with 10% tetracycline-free fetal calf serum (FCS), LCLs and EBV-negative BL2 Burkitt lymphoma B-cells (kindly provided by Elliott Kieff) were cultured in RPMI with 10% FCS. All cell lines were grown in a humidified ThermoFisher incubator at 37 C, with 5% CO2.

The following vectors were used in transient transfection assays: empty pSG5, pSG5 vectors with N-terminally FLAG-tagged GFP, TRAF1, TRAF2, or TRAF3 cDNAs; pSG5 expression vectors with untagged or N-terminally epitope-tagged full length LMP1 (wildtype), LMP1 residues 1–231, C-terminally HA-tagged LMP1 1–231; the TES2 null mutant _384_YYD_386_ ->_384_ID_385_, the TES1 null alanine point mutant LMP1 _204_PQQAT_208_-> _204_AQAAA_208_, or the TES1/TES2 null double mutant (LMP1 DM) containing both of these mutations [25,67]; a PGK-puro vector with untagged TRAF1 cDNA. N-terminally GST-tagged TRAF1 was cloned into a modified Gateway-compatible pSG5 expression vector by Gateway cloning.

For western blot analysis, antibodies against the following were used: Cell Signaling Technologies TRAF3 (#4729), cIAP1 (#7065), cIAP2 (# 3130), phospho-JNK (#9251), phospho-p38 (#9211), total JNK (#9252), total p38 (#9212), phospho-ERK (# 4377), total ERK (# 9102), RelA-phosphoserine 536 (3033); total RelA (8242) total Ub (3936), ABIN1 (4664), A20 (cat #4625); Bethyl Laboratories SHARPIN (#A303-559A), TRAF2 (#A303-460A), HOIP (A303-560A); Sigma Aldrich tubulin (# T5168) and FLAG M2; Santa Cruz TRAF1 (#sc1831) and NEMO (sc-8330); Covance HA.11; EMD Millipore p100/p52 (05–361) and anti-K63 05–1308; Chemicon/Millipore GAPDH (MAB375); Genentech anti-M1 Ub 1F11/3F5/Y102L. Detection of endogenous phospho-TAK1 was performed on inducible 1–231 LMP1 293 TRAF1 cells uninduced or induced for LMP1 expression overnight, and treated with 50 nM Calyculin A (Cell Signaling #9902) for 5 minutes prior to harvest. Cell Signaling antibodies against phospho-TAK1 (#4508) and total TAK1 (#4505) were used. LMP1 monoclonal antibodies OT22CN against the LMP1 N-terminus, or S12 against the LMP1 C-terminus (recognizes an epitope between TES1 and TES2), were used. Cell Signaling HRP-tagged secondary antibodies, or with Rockland TrueBlot HRP-tagged secondary antibodies were used for western blot.

### Western blot

All immune-purified and whole cell lysate samples were boiled for 5 minutes in Laemmli SDS-loading buffer with a final concentration of 1% SDS and 5% beta-mercaptoethanol, at 95°C for 5 min. SDS/PAGE was performed on Bio-Rad precast gels. 4–20% gradient gels were used for experiments with anti-ubiquitin western blots. Proteins were transferred to nitrocellulose filters for 1 hour at 100V using Bio-rad a power pack and minigel transfer apparatus. Blots were blocked with 5% non-fat dry milk for 30 minutes, then probed overnight with primary antibody at 4 degrees C, washed in TBST for 5 minutes x 4 cycles, incubated with secondary antibody for 1 hour, washed in TBST for 5 minutes x 4 cycles, developed with Western Lightening ECL developer, and imaged on a Carestream Molecular Imaging workstation. Where indicated, western blot band intensities were measured using Carestream software, using background subtracted net values.

### M1-pUb chain purification and western blot

M1-pUb and K63-pUb linked chains were purified under denaturing conditions, according to the manufacturer’s instructions. Briefly, for M1-pUb purification, cells were lysed in buffer containing 8M urea and 20mM Tris (pH 7.4), 135 mM NaCl, 1% Triton-X100, 10% glycerol, 1mM EDTA, and 1.5mM MgCl2, supplemented with Roche complete EDTA-free protease inhibitor tablet, 1 mM PMSF, 4mM 1 10 o-phenanthroline, sodium pyrophosphate, 10 mM-glycerophosphate, 2 mM sodium pyrophosphate, 1% aprotinin, and 2 mM N-ethylmaleimide, at RT for 10min. Insoluble debris was pelleted by 13,000 RPM microfuge, and urea concentration was then reduced to 7M by addition of lysis buffer. 2ug of M1-Ub antibody was added, and samples were rotated at RT overnight. Precipitate was pelleted by microcentrifuge, and then 20ul of Protein A sepharose beads (Invitrogen 101042) were added, and rotated for 2 hours at RT. Beads were washed 5X with 7M urea lysis buffer, with inhibitors. Western blots were preformed according to Genentech instructions, using wet transfer at 30V for 2 hours to nitrocellulose membranes. Primary antibody was added to blots for 1 hr at RT.

### NF-kB reporter assay

NF-kB activity was measured by a GFP reporter assay, as previously described [38]. Briefly, conditional LMP1 1–231 and LMP1-231 TRAF1 293 cells with a stably integrated NF-kB GFP reporter were used. LMP1 1–231 expression was induced for 20 hours by the addition of doxycycline and 4HT. NF-kB GFP reporter values were measured on a FACScalibur flow cytometer (BD Biosciences), and analyzed by Cell Quest software (BD Biosciences).

### SMAC mimetic treatment

The SMAC mimetic TL-32711 was obtained from Active Biochem (#A-1901), and used according to the manufacturer’s instructions at a concentration of 20 uM.

### RNAi analysis

293 inducible LMP1 cells were treated with Dharmacon/Thermo Fisher siRNAs for 72 hours prior to LMP1 induction, as previously described [38]. Briefly, LMP1 1–231 conditional 293 TRAF1 cells were reverse transfected with Dharmafect I lipid in 12-well plates. 72 hours later, LMP1 1–231 expression was induced by addition of 4HT and doxycycline, where indicated, for 16 hours. The non-targeting siRNA control (Catalogue # D-001810-10-20), and siGenome siRNAs against TRAF2, TRAF3, RNF31/HOIP, RBCK1/HOIL-1L and SHARPIN were used at a final concentration of 50 nM per siRNA pool. For GM12878 shRNA analysis, LCLs were transduced with VSV-G pseudotyped lentiviral vectors from the Broad Institute of Harvard and MIT RNAi consortium on day 0 and 1. Anti-GFP shRNA was used as a control. On day 2, LCLs were selected with puromycin (3 ug/ml), and analyzed at the indicated timepoints. Knockdowns were validated by western blot and qPCR analysis. All shRNA sequences are available upon request.

### CRISPR/Cas9 mutagenesis

GM12878 LCLs with stable *S*. *pyogenes* Cas9 expression were established by infection by lentiviral transduction and blasticidin selection, using pLentiCas9-Blast (Addgene plasmid # 52962). We verified that Cas9 was highly active in the selected LCL pool by transduction with a lentivirus that encodes GFP, and a sgRNA against GFP [68]. The PXPR-011 plasmid was kindly provided by John Doench, Broad Institute, and encodes GFP, as well as an sgRNA against GFP. PXPR-011 is therefore a convenient way to monitor Cas9 activity in cell lines. GM12878 cells transduced with PXPR-011 based lentivirus and selected with puromycin initially expressed GPF, but were then found to lose GFP expression in >85% of transduced cells (the residual 15% of cells that continue to express GFP despite sgRNA against GFP may be cells where the non-homologous end-joining pathway correctly repaired the Cas9-induced DNA double strand break) [68]. By contrast, nearly 100% of Cas9 negative GM12878 cells were GFP positive after transduction with the same lentivirus and puromycin selection. CRISPR single guide RNAs (sgRNA) targeting human RNF31 (which encodes HOIP) were designed using the online program CRISPRdirect (http://crispr.dbcls.jp/)[69], and the oligo GCCCTCAGCGGCCTCGGTAC was Synthesized by Life Technologies, cloned into the lentiGuide-Puro vector (Addgene plasmid # 52963), according to the protocol from the Zhang laboratory website (http://genome-engineering.org/)[70], and sequence verified. Lentiviruses encoding the HOIP sgRNA were constructed and used to transduce GM12878 Cas9+ cells. Transduced cells were selected by purmoycin. HOIP depletion efficiency was validated by western blot.

### Transient transfection

293 cell lines were transiently reverse transfected as previously described [38], using Effectene lipid (Qiagen). For most experiments, cells were transfected for 18 hours.

### Ubiquitin sensor analysis

293 cells were transiently transfected with the indicated plasmids (pSG5 LMP1, pSG5 FLAG-TRAF1, and/or the UBAN-GFP sensor [71,72]). UBAN-GFP is a fusion between the conserved linear Ubiquitin Binding domain of ABIN1 and NEMO/IKK-gamma and GFP. The UBAN-GFP biosensor has been validated to be highly specific for M1-pUb chains *in vitro* and *in vivo* [71,72]. 20 hours after transfection, cells were fixed, permeabilized, and stained where indicated with antibodies against LMP1 or TRAF1 (Santa Cruz, rabbit polyclonal). Secondary antibodies used were Alexa-561-conjugated anti-mouse and Alexa-633-anti-rabbit (both from Life Technologies). Cells were analyzed by confocal microscopy, and images were processed with Fiji (http://wiki.imagej.net/Fiji).

### In vitro ubiquitination assays

N-terminally GST-tagged TRAF1 expression vectors were constructed using Gateway cloning, and used for purification of recombinant TRAF1 from unstimulated HEK-293 cells. In vitro ubiquitin assays were performed as previously described [73]. Briefly, in vitro ubiquitination assays were performed according to the manufacturer’s protocol (Boston Biochem). Ubiquitin (5 μg), the E1 enzyme (200 ng), UBE2L3 (300 ng) (Boston Biochem), the indicated LUBAC components (0.8 μg) and TRAF1-GST(2μg) were co-incubated with 2 mM ATP (Sigma) at 37°C 2 hours, in ubiquitin assay buffer (20 mM Tris-HCl pH7.5, 5 mM MgCl2, 2 mM DTT). 1x stop solution (Boston Biochem) was added to terminate the reaction. Following GST pull-down, beads were washed four times, and then boiled in Laemmli SDS-loading buffer with 5% beta-mercaptoethanol at 95°C for 5 min. The samples were subsequently analyzed by SDS-PAGE followed by Western blotting using a PVDF membrane.

### Mass spectrometry analysis

FLAG affinity purification and liquid chromatography-mass spectrometry analysis were performed, as previously described[74]. Seven 15 cm^2 dishes (approximately 100 million cells) of 293 TRAF1 or GFP control cells, either uninduced or induced for LMP1 1–231 expression for 16 hours, were washed twice with PBS and then lysed on ice for 30 minutes in lysis buffer with protease inhibitors (Roche EDTA Free Complete, Cat #11836145001, 1% aprotinin (Sigma Cat #A6279), 1 mM PMSF (Sigma), and 4 mM 1,10 o-phenanthroline (Sigma), and the phosphatase inhibitors 10 mM beta-glycerophosphate and 2 mM sodium pyrophosphate (Sigma) [64]. 30 uL of packed anti-FLAG beads (Sigma Cat #A2220) were added into the lysates and were rotated at 4 degrees C for 4 hours, then washed 5 times in lysis buffer with protease and phosphatase inhibitors, with Eppendorf tube change prior to the last wash, and eluted with 50 ul of 3X-FLAG peptide (0.5 mg/ml, Sigma #F4799) at room temperature for 30 minutes, three times sequentially. Samples were analyzed by WB to confirm absence of antibody heavy/light chain contamination, and then run into a 10% pre-cast mini-gel (Bio-Rad) for 1 cm, cut into two equal slices, and sent for liquid chromatography mass spectrometry (LC/MS-MS) analysis at the Harvard Taplin Biological Mass Spectrometry Facility (Harvard Medical School). Gel slices were processed by the Taplin Proteomics facility staff. Briefly, gel slices were subjected to a modified in-gel trypsin digestion procedure. Gel pieces were washed and then dehydrated with acetonitrile for 10 min. Following acetonitrile removal, slices were speed-vac dried, rehydrated with a 50 mM ammonium bicarbonate solution containing 12.5 ng/μl modified sequencing-grade trypsin (Promega, Madison, WI) at 4°C. After 45 min., the excess trypsin solution was removed and replaced with 50 mM ammonium bicarbonate solution to just cover the gel pieces. Peptides were then extracted by removing the ammonium bicarbonate solution, followed by one wash with a solution containing 50% acetonitrile and 1% formic acid. Extracts were speed-vac dried for ~1 hr and stored at 4°C until analysis. On the day of analysis, samples were reconstituted in 5–10 μl of HPLC solvent A (2.5% acetonitrile, 0.1% formic acid), subjected to nano-scale reverse-phase HPLC using a capillary column (5 μm C18 spherical silica beads packed into a fused silica capillary (100 μm inner diameter x ~12 cm length) with a flame-drawn tip. After equilibrating the column, each sample was loaded via a Famos auto sampler (LC Packings) onto the column. A gradient was formed and peptides were eluted with increasing concentrations of solvent B (97.5% acetonitrile, 0.1% formic acid). Upon elution, peptides were subjected to electrospray ionization and analyzed by a LTQ Velos ion-trap mass spectrometer (ThermoFisher, San Jose, CA). Peptides were detected, isolated, and fragmented to produce a tandem mass spectrum of specific fragment ions for each peptide. Dynamic exclusion was enabled such that ions were excluded from reanalysis for 30 s. Peptide sequences (and hence protein identity) were determined by matching protein databases, using Sequest (ThermoFisher). The human IPI database (Ver. 3.6) was used for searching. Precursor mass tolerance was set to +/- 2.0 Da and MS/MS tolerance was set to 1.0 Da. A reversed-sequence database was used to set the peptide false discovery rate at 1%. Filtering was performed using the Sequest primary score, Xcorr and delta-Corr. Spectral matches were further manually examined.

### Interaction scoring for FLAG affinity purification-mass spectrometry (AP-MS) data

To assign statistical significance and to identify high-confidence TRAF1 interacting proteins, our data were compared with a publicly-available database of 30 negative control FLAG-tagged baits, purified from HEK-293 stable cell lines under similar conditions (http://www.crapome.org) [75]. Control dataset IDs and bait peptide counts are also provided for comparison in **S1 Table**. The SAINT algorithm (http://sourceforge.net/projects/saint-apms) was used to evaluate the MS data [76,77]. SAINT is designed for AP-MS analysis and has been validated in analysis of several protein interactomes [76,78,79]. The default SAINT options were low Mode = 1, min Fold = 0, norm = 0. SAINT probabilities computed independently for each biological replicate were averaged (AvgP) and reported as the final SAINT score. Fold change was calculated for each prey protein as the ratio of average spectral counts from replicate bait purifications over the average spectral counts across all negative controls (total peptide spectral counts were summed for each protein). A background factor of 0.1 was added to the average spectral counts of negative controls to prevent division by zero. The highest number of spectral counts for each protein were selected to establish the negative control database for SAINT analysis. Selection of the threshold for SAINT scores was based on receiver operating curve analysis performed using publicly available protein interaction data and the FLAG AP-MS data set as a list of true positive interactions. A SAINT score of AvgP ≥ 0.80 was considered a high-confidence interacting protein, with an estimated FDR of ≤1%.

### Real-time PCR

Real-time reverse transcription-PCR (qPCR). qPCR was performed on a Bio-Rad CFX Connect Real-time system, using the Power SYBR green RNA-to-CT 1-step kit (Applied Biosystems), for 40 cycles. Fold changes were determined using the CT method and normalized by 18S rRNA expression levels. The RBCK1 primers 5’-TGCAAGACCCCAGATTGCA-3’, AND 5’-ACAGGGCAGGTGAACTCATTG-3’ were used.

### Proliferation and apoptosis assays

96 hours after initial shRNA transduction (and 48 hours after puromycin selection), LCLs were plated at a density of 300,000 cells/ml in 96 well plates in 100 uL of RPMI/FCS, in triplicate. Cells were fed 100 uL of RPMI at 48 hours and 96 hours thereafter. Relative live cell numbers were then accurately quantitated by the CellTiter-Glo luminescent cell viability assay (Promega). All values obtained were within the linear range of the instrument. For shRNA growth curves, a LMaxII instrument was used (Molecular Devices). For CRISPR/cas9 growth curve analysis, SpectraMax L (Molecular Devices) was used, as the LMaxII was no longer available.

### Graphics

All bar-graphs and growth curves were produced using GraphPad software.

## Results

### TRAF1 enhances LMP1 1-231-mediated p38, ERK, JNK, and canonical NF-kB activation

**Fig 1:**
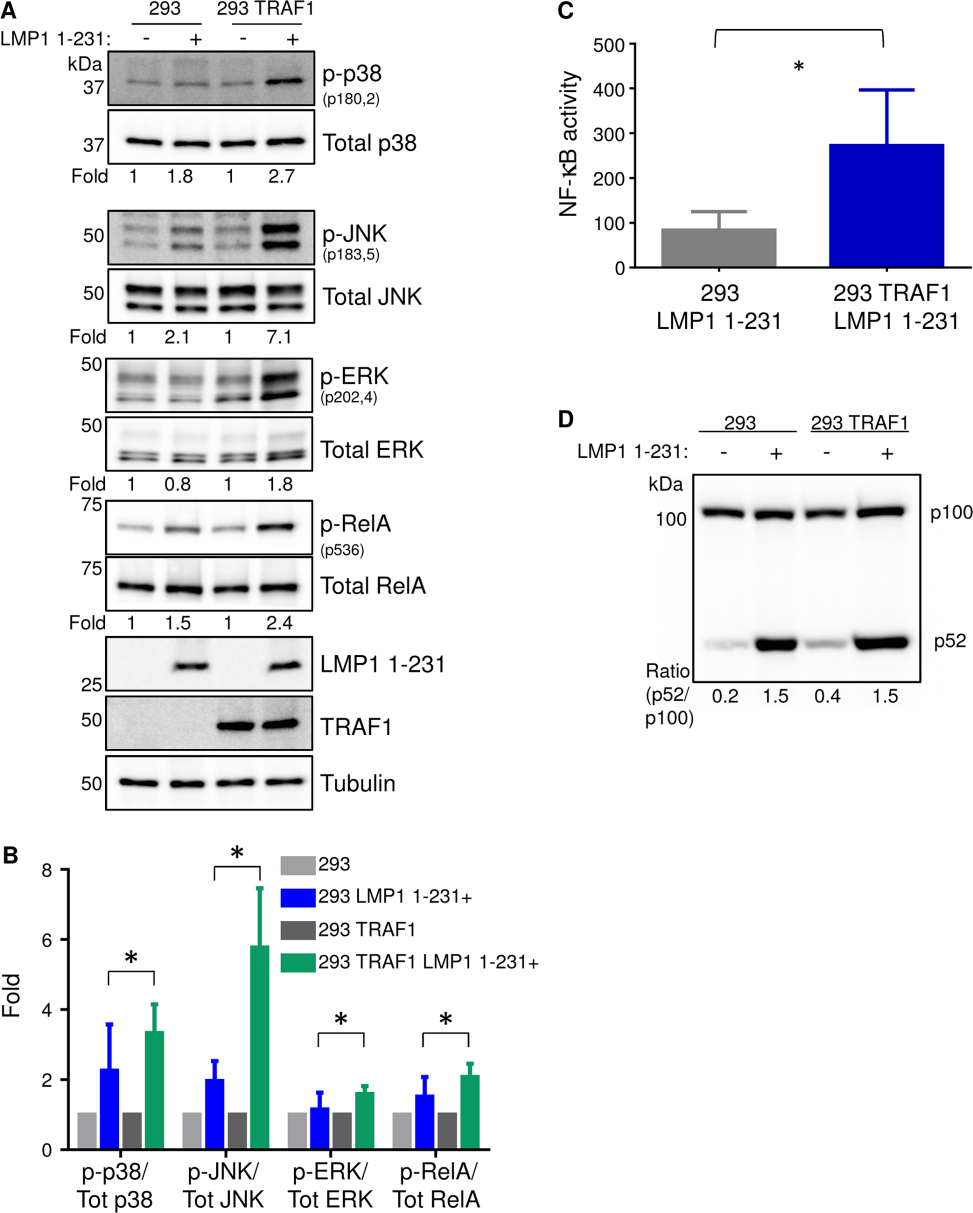
TRAF1 enhances LMP1 1-231-mediated MAP kinase and canonical NF-kB activation. A) 293 cells, and 293 cells that stably express TRAF1, were induced for LMP1 1–231 expression for 16 hours. Whole cell lysates were analyzed for p38, JNK and ERK activation by western blot (WB) analysis, using antibodies to their total and phosphorylated forms. Band intensities were quantitated, and the phosphoprotein:total protein ratio in uninduced cells was normalized to a value of 1 for each cell line. The phosphoprotein:total protein ratios are shown beneath each pair. B) Average and standard deviation fold changes from three independent experiments (including the blots presented in Fig 1A). Student’s 1-tailed T-test * P <. 05. C) LMP1 1–231 expression was induced for 16 hours in 293 or 293 TRAF1+ cells. NF-kB activity was measured by FACS, using a stably integrated GFP NF-kB reporter. Mean and standard deviation of three independent experiments are shown. D) Non-canonical NF-kB pathway activity was determined by WB analysis of p100/p52 processing in 293 or 293 TRAF1 cells, uninduced for LMP1 or following 16 hours of LMP1 1–231 induction. P100:p52 ratios are shown, and were representative of three independent experiments.

To characterize signaling by the LMP1 TES1 domain, 293 cells with conditional LMP1 1–231 expression were derived (described in detail in the Methods) [38,43]. We then established conditional LMP1 1–231 293 cell lines with stable expression of N-terminally FLAG- tagged GFP or TRAF1. FLAG-TRAF1 was expressed at LCL physiological levels (**S1 Fig**). The conditional 293 LMP1 cell pair provided an isogenic background with which to compare the effects of TRAF1 on LMP1 TES1 domain-mediated pathway activation. Consistent with prior LMP1 studies [29,52], TRAF1 co-expression markedly boosted LMP1 TES1-mediated JNK pathway activation (**Fig 1A** and **1B**). The ratio of phospho-JNK to total JNK in whole cell extracts increased from 2.1-fold in 293 cells to 7.1-fold in 293 TRAF1 cells 16 hours after LMP1 1–231 induction, as judged by western blot analyses from three independent experiments. We likewise found that TRAF1 co-expression significantly increased LMP1 1-231-mediated p38 and ERK phosphorylation (**Fig 1A** and **1B**). Our results suggest that TRAF1 enhances LMP1 TES1 domain-mediated activation at a level upstream of the three MAP kinases.

Consistent with a prior report, we found that TRAF1 co-expression also significantly enhanced LMP1 TES1-mediated NF-kB activation (**Fig 1C**) [29]. We next examined whether TRAF1 co-expression affected LMP1 1-231-mediated canonical and/or non-canonical NF-kB pathway activation. TRAF1 co-expression significantly up-regulated LMP1 TES1-mediated canonical NF-kB, as judged by RelA serine 536 phosphorylation, a commonly used marker of canonical NF-kB activity (**Fig 1A** and **1B**). To examine the effect of TRAF1 co-expression on LMP1 1-231-mediated non-canonical NF-kB activation, we analyzed the p100:p52 ratio in conditional 293 and 293 TRAF1 cells. Non-canonical NF-kB activity triggers proteasomal processing of p100 to p52. Interestingly, TRAF1 co-expression did not significantly enhance LMP1 1-231-mediated non-canonical NF-kB pathway activity, as judged by the ratio of p100:p52, which remained 1.5 in both conditions (**Fig 1D**). Since the kinase TAK1 plays key roles in LMP1 MAP kinase and canonical NF-kB pathways, we next tested whether TRAF1 enhanced LMP1 1-231-mediated TAK1 activation. Indeed, TRAF1 markedly enhanced LMP1 1–231 induction of TAK1 activation loop serine 187 phosphorylation (**S2 Fig**). Collectively, our results suggest that TRAF1 enhanced LMP1 1-231-mediated MAP kinase and canonical NF-kB pathway activation at or above the level of TAK1 activation.

### LMP1 1–231 increases association between TRAF1 and LUBAC

To gain insights into TRAF1 effects on LMP1 TES1 signaling, we used affinity purification and mass spectrometry analysis (AP-MS) of TRAF1 complexes as a discovery tool for subsequent analysis. FLAG-TRAF1 complexes were immune-purified from conditional 293 cells that were uninduced or induced for LMP1 1–231 expression for 16 hours [64]. Complexes were eluted from agarose beads by co-incubation with FLAG peptide (see **Methods** section for details). As a negative control, FLAG-GFP was immuno-purified from conditional LMP1 1–231 cells induced for 16 hours. Independent FLAG-purifications were analyzed by liquid chromatography-mass spectrometry/mass spectrometry (LC-MS/MS) proteomic analysis for each condition. Data resulting from AP-MS analysis are presented in **S1 Table**.

To identify high-confidence interactions in our TRAF1 datasets, we further compared our datasets with thirty publically-available 293 cell FLAG AP/MS control datasets (http://www.crapome.org)[75]. This analysis provides additional statistical power to remove common 293 cell contaminants, which are frequently high abundance proteins including heat-shock, cytoskeletal, histones, ribonucleoproteins, and ribosomal proteins. We used the well-established ‘Significance Analysis of Interactome' (SAINT) computational algorithm to assign confidence scores to our TRAF1 datasets [76,77,80]. SAINT uses quantitative AP-MS data to derive the probability of a bona fide protein-protein interaction. At a FDR < 1% cutoff (SAINT score ≥0.8), we identified 19 high-confidence TRAF1 interactors in extracts from LMP1 1–231 expressing 293 cells. Three additional proteins had a SAINT score of 0.79 and were also considered high-confidence interactors. 23 high-confidence TRAF1 interactors were identified from uninduced 293 cells purifications (**S3 Fig and S1 Table)**. Well-characterized TRAF1 interactors were enriched in both TRAF1 datasets, including TRAF2, cIAP1, cIAP2, TBK1, and TANK. Other TRAF1 high confidence interactors were identified in either the LMP1 1–231 uninduced or induced condition. Interestingly, all seven of the LMP1 1-231-induced TRAF1 high-confidence protein interactions have established roles in ubiquitin biology, including ubiquitin itself. Notably, LMP1 1–231 expression induced association between TRAF1 and the LUBAC catalytic subunits HOIP (SAINT score 0 without induction, 0.99 with induction) and HOIL-1L (SAINT score 0 without induction, 0.79 with induction). By comparison, HOIP or HOIL-1L peptides were not retrieved in our FLAG-GFP samples or in any of the 30 control 293 cell FLAG runs (**S1 Table**). LMP1 1–231 expression also induced TRAF1 association with the ubiquitin editor protein A20 (SAINT score 0 without induction, 0.96 with induction). A20 contains deubiquitinase, ubiquitin ligase, and ubiquitin-binding zinc finger domains, the latter of which bind to both M1- and K63-pUb chains. The K63-polyubiquitin binding protein SQSTM1/P62 was also identified as a high-confidence LMP1-induced TRAF1 interactor (uninduced SAINT score 0, induced score 0.79). The K63- and M1-pUb sensor ABIN1 nearly reached significance (SAINT score without induction, 0.49 with induction).

### Validation of TRAF1 and LUBAC association in cells that express LMP1

**Fig 2:**
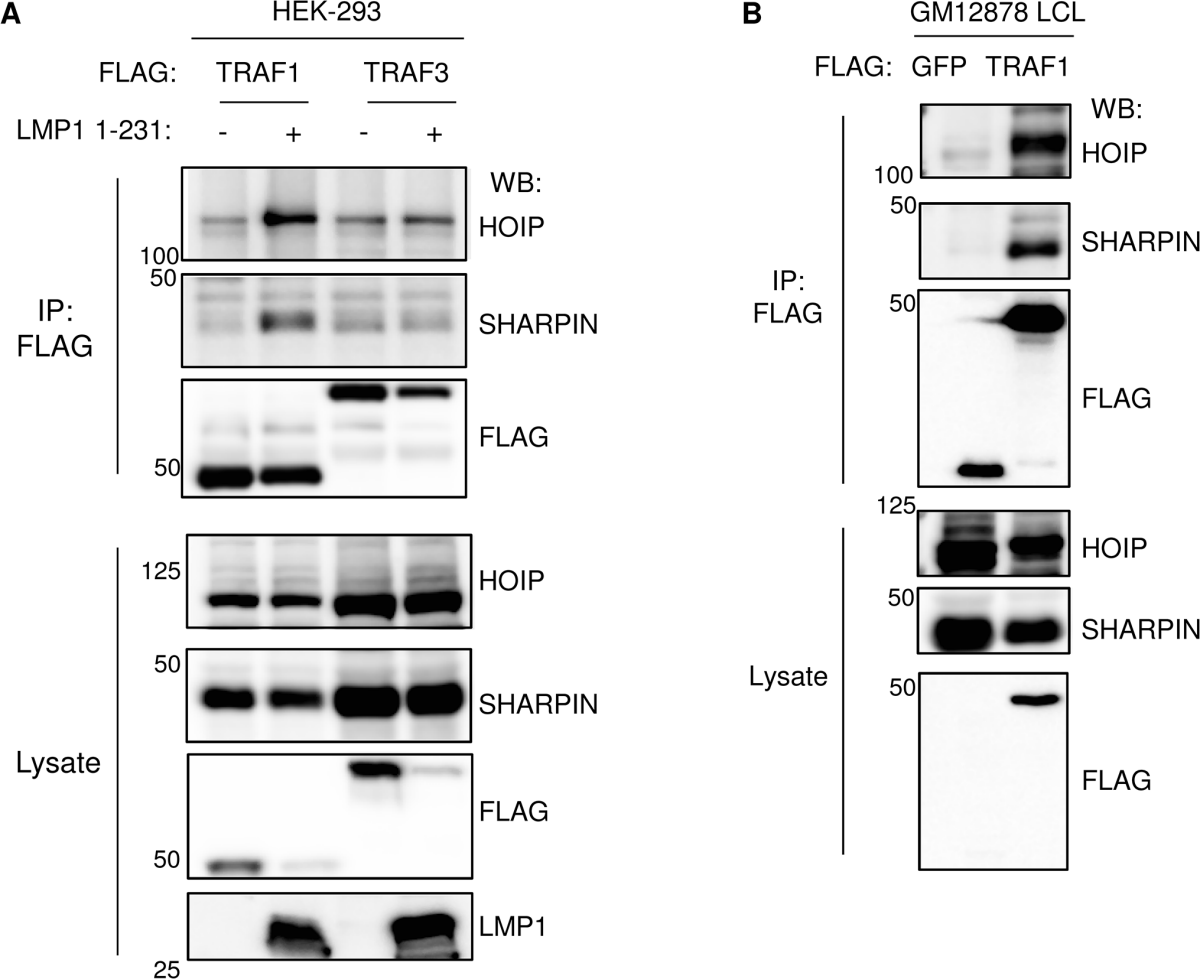
TRAF1 and LUBAC associate in LMP1-stimulated cells. A) 293 cells were transfected with FLAG-tagged TRAF1 or TRAF3, and LMP1 1–231 expression was induced 24 hours, as indicated. FLAG immuno-purified complexes and lysates were analyzed by western blot (WB), as indicated. B) FLAG immuno-purified complexes and lysates were immuno-purified GM12878 LCLs with stable FLAG-GFP or FLAG-TRAF1 expression, and analyzed by western blot, as indicated. A-B are representative of three independent experiments.

The LMP1 1-231-induced association between TRAF1 and LUBAC was further validated by IP/western blot analysis, using both conditional 293 cells and GM12878 LCLs with stably FLAG-TRAF1 expression at physiological levels (**S1 Fig**). First, FLAG-TRAF1 complexes were immuno-purified from conditional 293 cells, either undincued or induced for LMP1 1–231 expression for 16 hours (**Fig 2A**). LMP1 induction increased the level of HOIP and SHARIPN in FLAG-TRAF1 complexes. By contrast, we did not observe significant co-purification of LUBAC components with FLAG-TRAF3 complexes immuno-purified from conditional LMP1 1–231 293 cells with stable FLAG-TRAF3 expression. Of note, twice as many FLAG-TRAF3 cells were used in this experiment, to achieve similar levels of immuno-purified FLAG-TRAF1 and FLAG-TRAF3. We were unable to find a suitable antibody for analysis of endogenous HOIL-1L. Also of note, LMP1 1–231 induction caused TRAF1 and TRAF3 steady state levels to decrease, perhaps as a result of increased turnover. To validate that TRAF1 associates with LUBAC in LCL extracts, we tested whether FLAG-TRAF1 purified from GM12878 cells also retrieved LUBAC components. Both HOIP and SHARPIN co-immunoprecipitated with FLAG-TRAF1, but not with a FLAG-GFP control (**Fig 2B**). Likewise, HA-SHARPIN complexes immuno-purified from GM12878 cells with stable HA-SHARPIN expression co-immunoprecipitated TRAF1 and LMP1 (**S4 Fig)**. Taken together, our data suggest that TRAF1 and LUBAC are present together in protein-protein complexes in LMP1+ cells.

### LMP1 and TRAF1 complexes are modified by linear ubiquitin chains in LCLs

**Fig 3:**
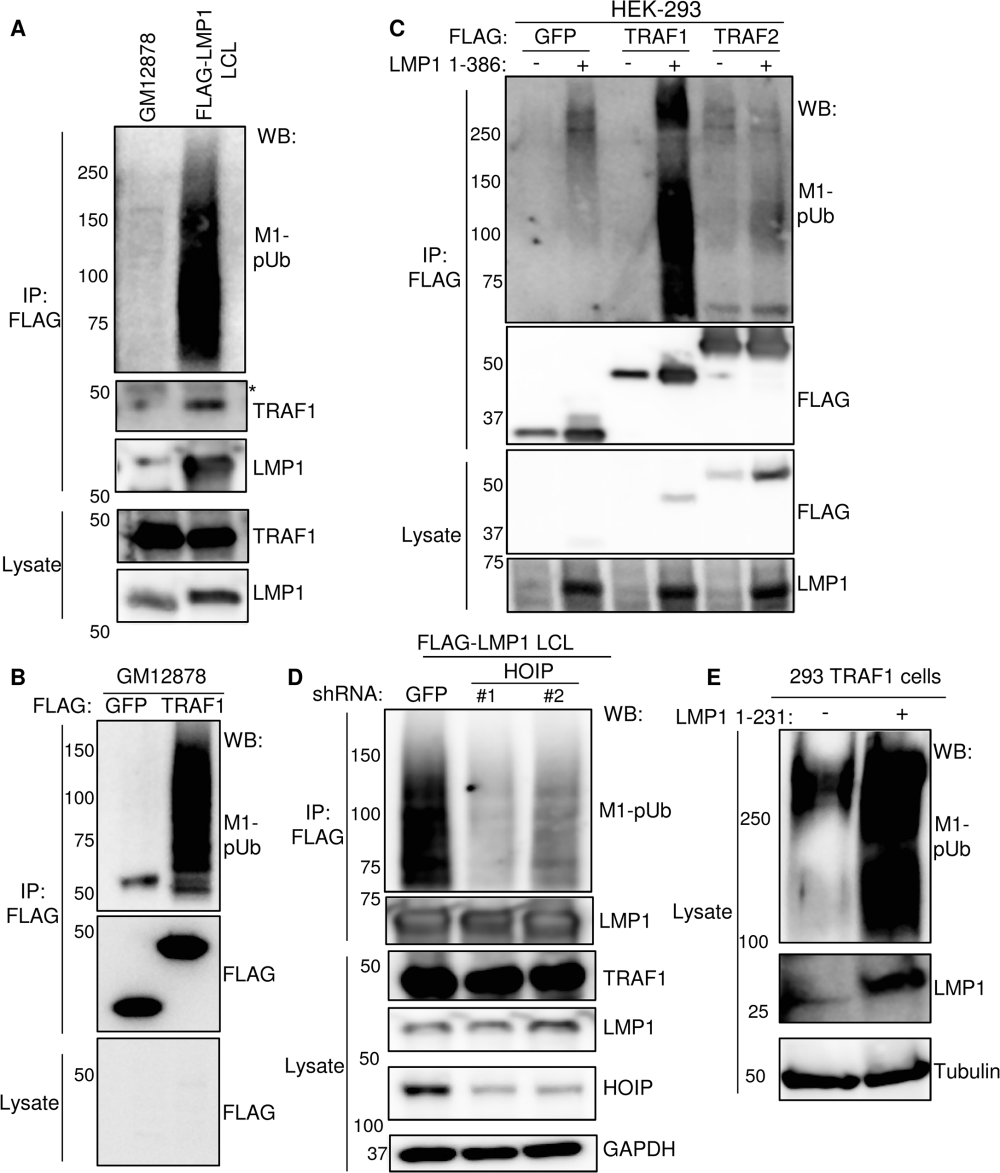
LMP1 and TRAF1 complexes are modified by M1-pUb chains. A) FLAG-LMP1 complexes were immuno-purified from LCLs that express FLAG-LMP1 from the EBV genome at physiologic levels [18]. GM12878 LCLs were used as a negative control. M1-linked polyubiquitin chain (M1-pUb) content was analyzed by WB, using a highly chain specific antibody. * indicates antibody heavy chain background. FLAG-LMP1 migrates at a slightly higher than untagged LMP1. B) FLAG complexes were immuno-purified from GM12878 LCLs that stably express either FLAG- GFP or FLAG-TRAF1, and were immuno-blotted, as indicated. C) 293 cells were co-transfected with FLAG- GFP, FLAG-TRAF1, or FLAG-TRAF2, and either empty PSG5-vector control or untagged LMP1 1–386 for 24 hours. Purified FLAG complexes or whole cell lysates were blotted for FLAG or LMP1, as indicated. D) FLAG-LMP1 LCLs were transduced with lentiviruses that express control shGFP or an independent shRNA against HOIP. Following puromycin selection, FLAG LMP1-immuno-purified complexes and lysates were blotted, as indicated. E) Whole cell extracts from 293 TRAF1 cells, uninduced or induced for LMP1 1–231 expression for 16 hours, were blotted for M1-pUb, LMP1, or tubulin. A-E are representative of three independent experiments.

Most TRAF1 in LCLs is associated with LMP1 [29]. We therefore investigated whether LMP1 complexes are modified by M1-linked pUb chains in LCL extracts. FLAG-LMP1 was immuno-purified from LCLs established from recombinant EBV, in which FLAG-tagged LMP1 is expressed at physiological levels from the EBV genome [18]. As a negative control with physiologic LMP1 expression from the EBV genome, we used GM12878 LCLs (where LMP1 is untagged). FLAG immuno-purified material was subjected to western blot analysis, using a M1-linked pUb (M1-pUb) chain specific monoclonal antibody [81]. M1-pUb chains were readily detected in FLAG-LMP1 purified from FLAG-LMP1 LCL extracts, but not from GM12878 extracts, suggesting that either LMP1, or an LMP1 signalosome protein, was modified by LUBAC-catalyzed M1-pUb chains (**Fig 3A**).

We next investigated whether TRAF1 complexes purified from GM12878 LCLs were likewise decorated with M1-pUb chains. As a negative control, we used GM12878 that express N-terminally FLAG-tagged GFP at similar levels. As shown in **Fig 3B,** FLAG-immuno-purified TRAF1 complexes, but not FLAG-GFP complexes, were highly modified by M1-pUb chains. Thus, LMP1 and TRAF1 are each present in complexes that are highly modified by M1-pUb chains.

To determine whether LMP1 induces M1-pUb chain attachment to TRAF1 complexes, we used 293 cell transient transfection assays. 293 cells were co-transfected for 24 hours with N-terminally FLAG-tagged GFP, TRAF1 or TRAF2 and either empty vector or untagged wildtype LMP1. We used the transfection system rather than the conditional system for this analysis to achieve similar FLAG-tagged protein expression levels across all three baits. FLAG immuno-purified complexes were analyzed by western blot for M1-pUb chains. Interestingly, FLAG-TRAF1 complexes, retrieved from LMP1 co-transfected cells, were highly modified by M1-pUb chains (**Fig 3C**). By contrast, background levels of M1-pUb chains were observed in FLAG-GFP and FLAG-TRAF2 pulldowns, and in FLAG-TRAF1 pulldown from cells without LMP1. This result suggests that LMP1 stimulates M1-pUb chain attachment to TRAF1, or a TRAF1-associated protein, and that in the absence of TRAF1, LMP1 does not induce M1-pUb attachment to TRAF2 complexes.

To next determine whether HOIP is important for M1-pUB chain attachment to LMP1 complexes in LCLs, we depleted HOIP from FLAG-LMP1 LCLs, using two independent shRNAs. Each anti-HOIP shRNA, but not the anti-GFP shRNA control, markedly reduced M1-pUb chain decoration of FLAG-LMP1 complexes, suggesting that HOIP plays a key non-redundant role in M1-chain attachment to LCL TRAF1 complexes (**Fig 3D**). Finally, we investigated whether LMP1 1–231 expression stimulates LUBAC activity. Whole cell lysates from 293 TRAF1 conditional cells that were uninduced, or induced for LMP1 1–231 for 16 hours, were analyzed for M1-pUb chain content by western blot (**Fig 3E**). Extracts from induced cells had abundant immune-reactive material. Of note, similar M1-pUb chain formation in 293 cells was previously demonstrated by HOIP/HOIL-1L transient transfection [81].

### LMP1 stimulates association between TRAF1 and ubiquitin sensors

**Fig 4:**
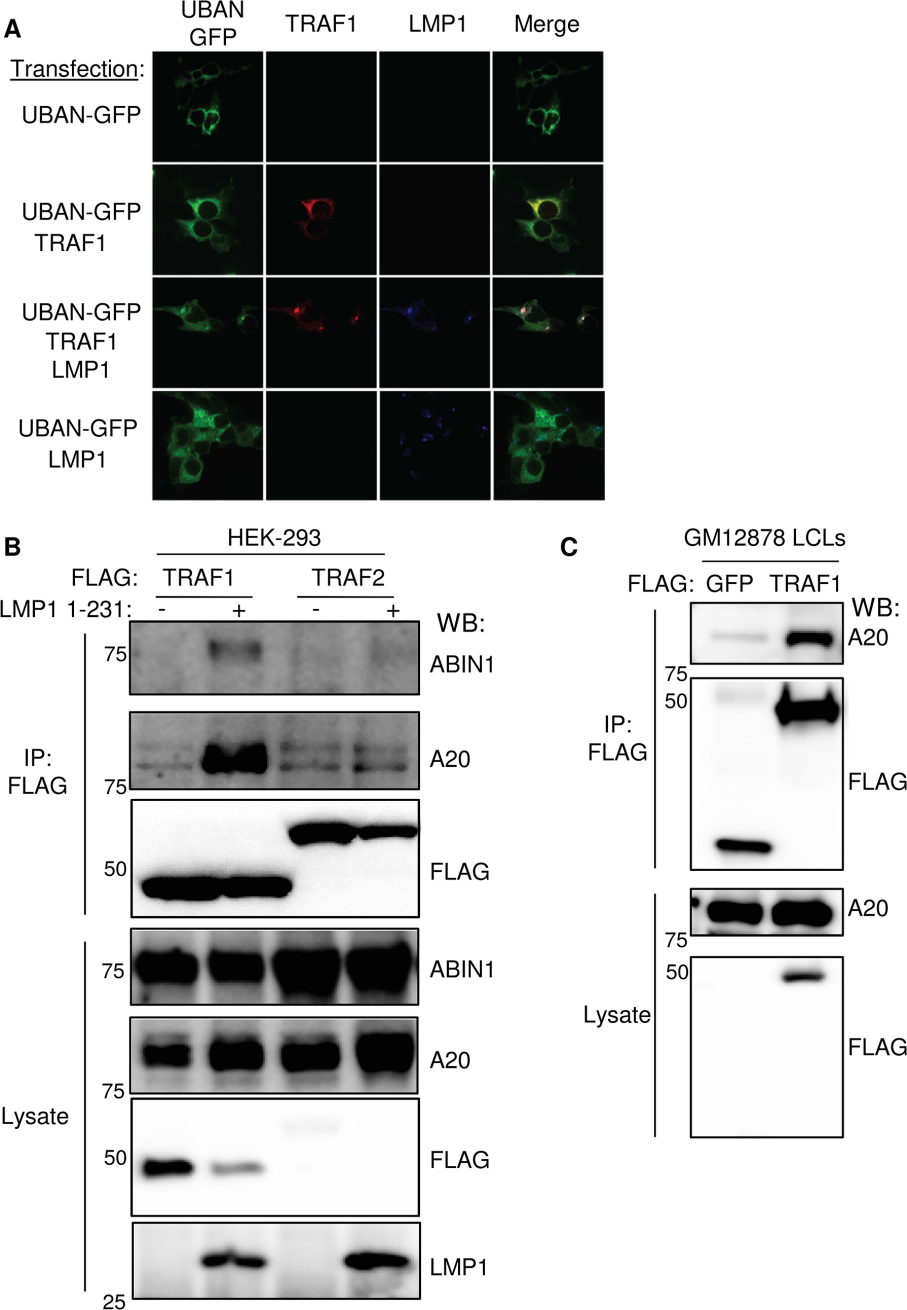
LMP1 induces TRAF1 association with M1-pUb chain sensors. A. 293 cells were transfected with LMP1, FLAG-TRAF1 and/or UBAN-GFP (a fusion protein that contains the IKK-gamma M1-pUb binding domain and GFP), as indicated. 24 hours later, cells were stained for TRAF1 and LMP1, and imaged by confocal microscopy. Additional confocal images are shown in **S5B Fig**) 293 cells were transfected with FLAG-TRAF1 or TRAF2, and after 24 hours, induced for LMP1 1–231 expression for 16 hours. FLAG immuno-purified material and lysates were blotted, as indicated. C) FLAG immuno-purified complexes and lysates from stable GM12878 FLAG-GFP or FLAG-TRAF1 cells were immuno-blotted, as indicated. A-C are representative of three independent experiments.

To further establish that LMP1 and TRAF1 complexes are decorated by M1-pUb chains *in vivo*, we next tested whether LMP1 and TRAF1 co-localize with a M1-pUb chain biosensor. The biosensor is comprised of a fusion protein between GFP and the Ubiquitin Binding of ABIN1 and NEMO/IKK-gamma (UBAN) domain. The UBAN-GFP biosensor selectively visualizes the localization of M1-pUb chains in mammalian cells activated by multiple independent stimuli [71,72]. 293 cells were transiently co-transfected with UBAN-GFP, TRAF1 and or/ LMP1. The characteristic 293 cell LMP1 punctate staining pattern was observed by confocal microscopy analysis of LMP1-transfected cells. By contrast, TRAF1 and UBAN-GFP exhibited diffuse cystosolic staining patterns in the absence of LMP1 co-expression (**Fig 4A**). Interestingly, LMP1 co-expression with TRAF1 and UBAN-GFP altered the TRAF1 and UBAN-GFP patterns, and induced marked co-localized of all three into punctate foci (**Figs 4A and S5**). We did not observe similar punctate foci of UBAN-GFP or co-localization with LMP1 in the absence of TRAF1 co-expression. Of note, TRAF1 and the UBAN-GFP sensor colocalized to a lesser extent, even in the absence of LMP1. Taken together with the proteomic and biochemical data presented above, these results are further suggest that in cells with LMP1 and TRAF1 co-expression, LMP1 and TRAF1 are present in complexes modified by M1-pUb chains.

We next tested whether TRAF1 and IKK-gamma also associate in GM12878 LCL extracts. Using GM12878 cells that stably express FLAG-tagged TRAF1 or FLAG-GFP as a control, we found that endogenous IKK-gamma co-immunoprecipitated with FLAG-TRAF1, but not with the FLAG-GFP negative control (**S6 Fig**). Likewise, using stable GM12878 cell lines, we found that endogenous TRAF1 reciprocally co-immunoprecipitated with HA-tagged IKK-gamma, but not with the HA-GFP negative control (**S6 Fig**). Of note, IKK-gamma expression in LCLs is significantly higher than in 293 cells, perhaps explaining why the 293 cell TRAF1 AP/MS analysis did not also identify IKK-gamma as a high confidence TRAF1 interactor, given the limit of detection of the assay. Collectively, these results are consistent with a model in which M1-pUb-linked pUb chains attached to TRAF1 complexes recruit the IKK-gamma.

We further validated the AP/MS result that LMP1 1–231 expression induced association between TRAF1 and the M1- and K63-pUb sensors A20 and ABIN1. A20 and ABIN1 are feedback regulators that each contain M1- and K63-pUb binding domains A20 down-modulates LMP1 TES1 signaling [82]. We found that immuno-purified FLAG-TRAF1 co-immunoprecipitated A20 and ABIN1 in 293 cells induced for LMP1 1–231 expression for 16 hours. By contrast, FLAG-TRAF2 complexes, purified from conditional LMP1 1–231 cells that stably express FLAG-TRAF2, did not co-immunoprecipitate A20 or ABIN1, even 16 hours after LMP1 1–231 induction (**Fig 4B**). Consistent with the higher A20 SAINT score in our TRAF1 AP/MS analysis, A20 association with TRAF1 complexes appeared to be more robust than that of ABIN1 by western blot analysis. Also of note, LMP1 expression increased A20 expression levels, as has previously been reported. Finally, endogenous A20 co-immunoprecipitated with FLAG-TRAF1, immuno-purified from GM12878 extracts (**Fig 4C**).

### Role of the LMP1 TES1 PQQAT motif and the TRAF1 coiled-coil domain in M1-pUb chain attachment

**Fig 5:**
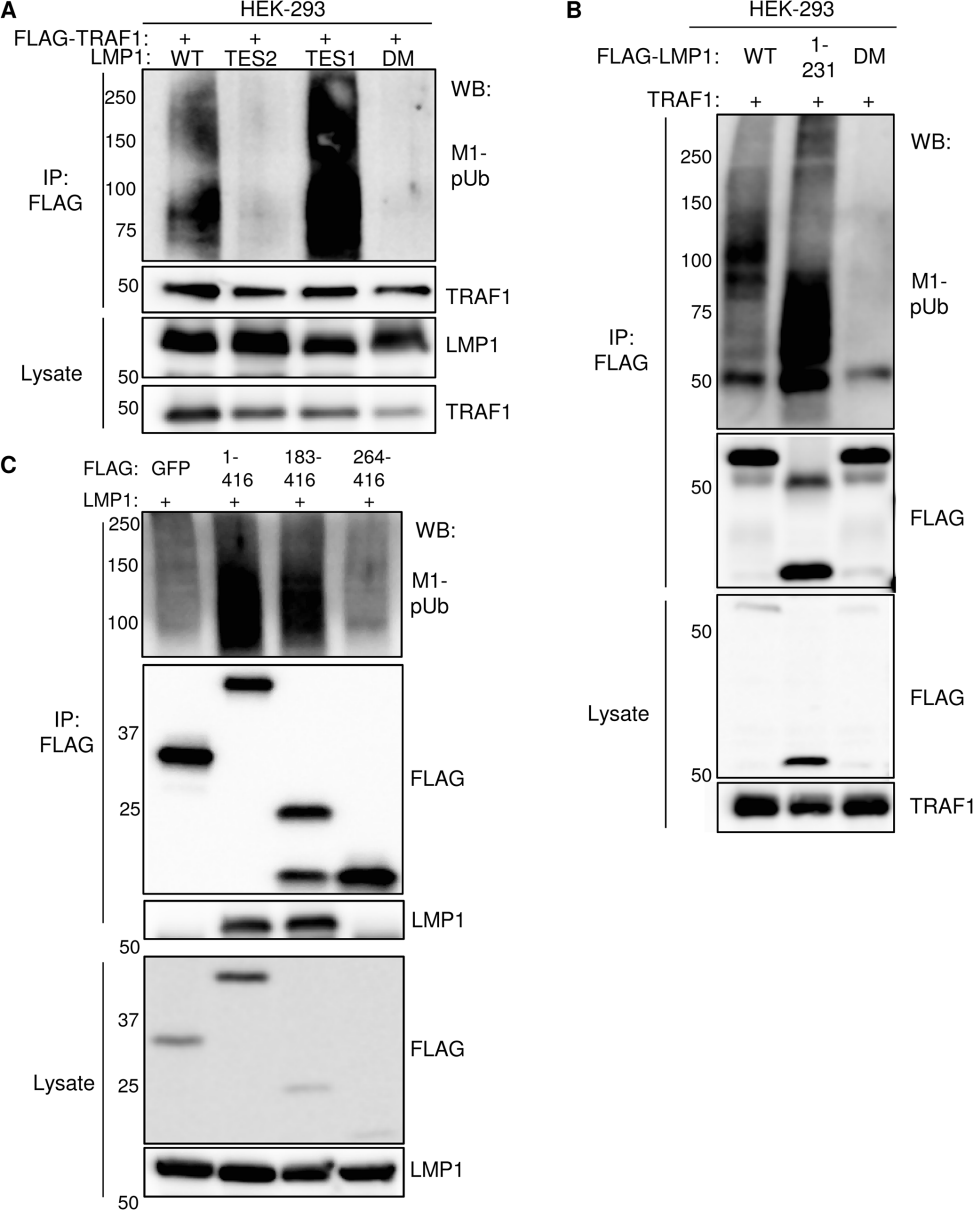
Analysis of LMP1 and TRAF1 domains important for M1-pUb attachment. A) 293 cells were co-transfected with FLAG-TRAF1, and with either wildtype LMP1 (WT), an LMP1 mutant that signal only from the TES2 domain (TES2), an LMP1 mutant that signals only from the TES1 domain (TES1), or an LMP1 double mutant (DM) that does not signal from either TES1 or TES2. Immuno-purified FLAG-TRAF1 complexes and whole cell lysates were blotted, as indicated. B) 293 cells were transfected with FLAG-tagged WT LMP1, LMP1 1–231, or LMP1 DM and untagged TRAF1. Purified FLAG complexes or lysates were blotted as indicated. C) 293 cells were co-transfected with untagged LMP1 and FLAG-tagged GFP, TRAF1 1–416, TRAF1 183–416, or TRAF1 264–416. FLAG complexes or lysates were blotted, as indicated. A-C are representative of triplicate experiments.

We previously found that HOIP and HOIL-1L are important for LMP1 TES2-mediated canonical NF-kB pathway activation in a genome-wide siRNA screen [38], suggesting that LMP1 TES2 may also activate LUBAC activity. To determine whether signaling by LMP1 TES2 also stimulates addition of M1-pUb chains to TRAF1 complexes, 293 cells were co-transfected with either wildtype (WT) LMP1, or with LMP1 mutants deficient for TES1, TES2 or TES1/TES2 signaling and with FLAG-tagged TRAF1. M1-pUb chains were detectable on FLAG-TRAF1 complexes immuno-purified from cells that co-expressed wildtype LMP1, or the LMP1 _384_ID_385_ mutant, which is null for TES2 signaling. By contrast, FLAG-TRAF1 complexes purified from cells that co-expressed either a LMP1 TES1 null alanine point mutant (LMP1 _204_PQQAT_208_-> _204_AQAAA_208_) deficient for TRAF recruitment or a LMP1 double mutant (DM) deficient for TES1 and TES2 signaling, were not modified by M1-pUb chains (**Fig 5A)**. These results suggest that association between TRAF1 and the LMP1 TES1 domain are required for M1-pUb chain attachment to TRAF1 complexes, and that LMP1 TES2-mediated NF-kB activation does not stimulate LUBAC to modify TRAF1.

We next determined whether LMP1 TES1 signaling was important for M1-pUb chain attachment to LMP1 complexes. 293 cells were co-transfected with FLAG-tagged WT, 1–231, or DM LMP1 vectors and with untagged TRAF1. FLAG-LMP1 immuno-purified complexes were analyzed by western blot for M1-pUb chain attachment (**Fig 5B**). M1-pUb chains decorated WT and LMP1 1–231 complexes, but not DM LMP1 complexes. These results again suggest that LMP1 complexes are modified by M1-pUb chains in cells with TRAF1 expression, and that LMP1 TES1 signaling is important for M1-pUb chain attachment to LMP1 complexes.

To identify TRAF1 domains important for LMP1-induced M1-pUb attachment, 293 cell transient transfection assays were performed with FLAG-tagged TRAF1 constructs, co-transfected with untagged WT LMP1. 24 hours after transfection, FLAG immuno-purified complexes were analyzed by western blot. Interestingly, FLAG-TRAF1 183–416 complexes, but not FLAG-TRAF1 264–416 complexes expressed at a similar level, were modified by M1-pUb chains (**Fig 5C**). TRAF1 183–264 residues form a coiled-coil (CC) domain, which is required for the formation of TRAF homo- and hetero-trimers. Notably, FLAG-TRAF 264–416 did not associate with LMP1, suggesting that TRAF1 trimerization and/or physical association with LMP1 are important for incorporation into complexes that contain M1-pUb chains. This result is consistent with the prior observation that TRAF trimers, rather than monomers, associate tightly with activated CD40 receptors.

### TRAF2 and the LUBAC subunits HOIP, HOIL-1L and SHARPIN, but not TRAF3 or cIAP1/2, are important for M1-pUb attachment to TRAF1 complexes

**Fig 6:**
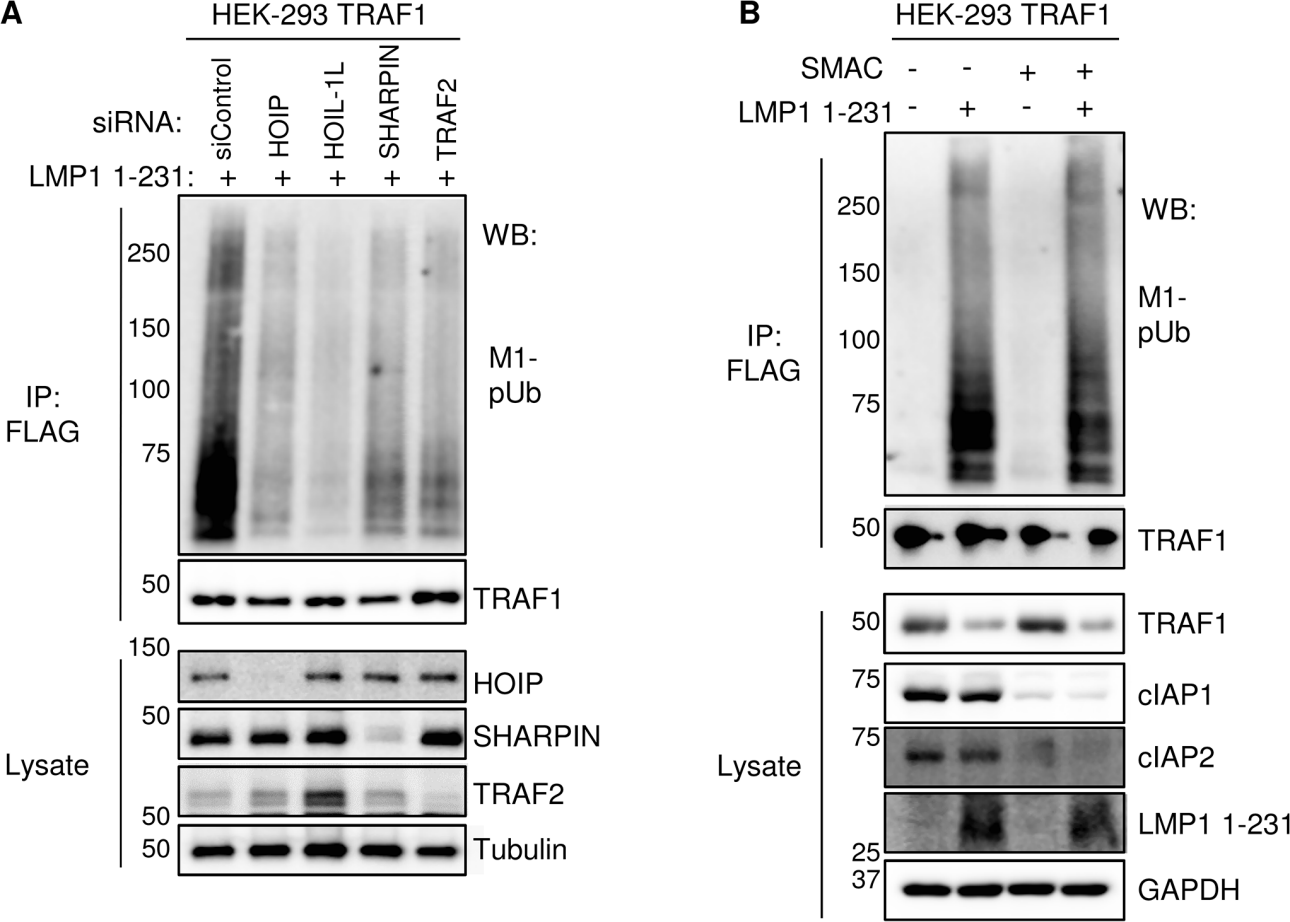
TRAF2, HOIP, HOIL-1L, and SHARPIN, but not cIAP1/2, are important for LMP1 1-231-induced M1-pUb chain attachment to TRAF1 complexes. A) 72 hours after siRNA transfection of 293 TRAF1 cells, LMP1 1–231 expression was induced for 16 hours. FLAG-TRAF1 complexes and lysates were WB, as indicated. B) 293 TRAF1 cells were treated with a SMAC mimetic peptide to deplete cells of cIAP ligases, and were then induced for LMP1 1–231 expression in the presence of the SMAC mimetic, as indicated. FLAG-TRAF1 IPs and lysates were blotted as indicated. A-B are representative of three independent experiments.

Loss-of-function approaches were used to test the importance of LUBAC subunits, TRAF2, TRAF3 and cIAP1/2 in M1-pUb chain attachment to TRAF1 complexes, since each were identified as high-confidence TRAF1 interactors. First, we used an siRNA approach to investigate the role of the three LUBAC components. 72 hours after 293 TRAF1 cell siRNA transfection, LMP1 1–231 expression was induced for 16 hours. The M1-pUb chain content of FLAG- TRAF1 immuno-purified complexes was analyzed by western blot. Knockdown efficiency was measured by western blot, using whole cell lysates (**Figs 6A and S7).** We were unable to identify commercially available antibodies that recognized endogenous HOIL-1L in our 293 cells, and instead used quantitative PCR analysis to validate HOIL-1L mRNA depletion in a parallel experiment (**S8 Fig**). Interestingly, we found that depletion of HOIP, HOIL-1L, or SHARPIN each impaired M1-pUb chain attachment to TRAF1 complexes, suggesting that all three LUBAC components play important and non-redundant roles, at least in 293 TRAF1 cells (**Fig 6A**). TRAF2 depletion likewise reduced M1-pUb chain abundance in purified FLAG-TRAF1 complexes, and also diminished association between TRAF1 and the LUBAC components HOIP and SHARPIN (**Figs 6A and S7**). By contrast, TRAF3 depletion did not impair M1-pUb chain attachment to purified TRAF1 complexes (**S7 Fig**). Taken together with our prior observation that TRAF2 complexes are not modified by M1-pUb in cells that lack TRAF1 expression, our results suggest that a TRAF1:TRAF2 heterotrimer, rather than a TRAF1 homotrimer, may be the functional unit that associates with LUBAC. Of note, HOIL-1L knockdown increased TRAF2 steady state levels (**Fig 6A**), while HOIP and SHARPIN knockdown also increased TRAF2 levels to a lesser extent. To our knowledge, LUBAC has not previously been implicated in control of TRAF2 steady-state levels, though it remains possible that this effect is specific to cells that express LMP1. Depletion of HOIP and HOIL-1L from 293 TRAF1 cells impaired LMP1 1-231-mediated p38, JNK and canonical NF-kB activation, consistent with a role for M1-pUB chains at or above the level of TAK1 kinase activation (**S9 Fig**).

Given the key roles that TRAF2 and cIAP ligases play in TNFR1-mediated LUBAC recruitment and activation, and since the TRAF1:TRAF2 heterotrimer more efficiently recruits cIAP ligases than TRAF2 homotrimers [83], we next tested the importance of cIAP1 and cIAP2 in M1-pUb chain attachment to TRAF1 complexes. cIAP1 and cIAP2 perform largely redundant functions, potentially complicating siRNA loss-of-function approaches that would require their compound knockdown. We therefore used a cell-permeable SMAC mimetic peptide to deplete cIAP1 and cIAP2. SMAC mimetics induce cIAP1/2 auto-ubiquitination and rapid proteasomal degradation [84]. Interestingly, 293 TRAF1 cell treatment with SMAC mimetic prior to and throughout the 16 hours of LMP1 1–231 expression did not impair subsequent attachment of M1-pUb chains to LMP1/TRAF1 complexes, despite efficiently inducing cIAP1/2 depletion from 293 cell whole cell lysates (**Fig 6B**). This result suggests that LMP1-induced LUBAC association with TRAF1:TRAF2 complexes differs from TNF-alpha induced LUBAC recruitment to TRAF2:cIAP complexes, which require cIAP-catalyzed pUb chain formation.

### LMP1 and TRAF1 as targets of M1-pUb chain attachment

**Fig 7:**
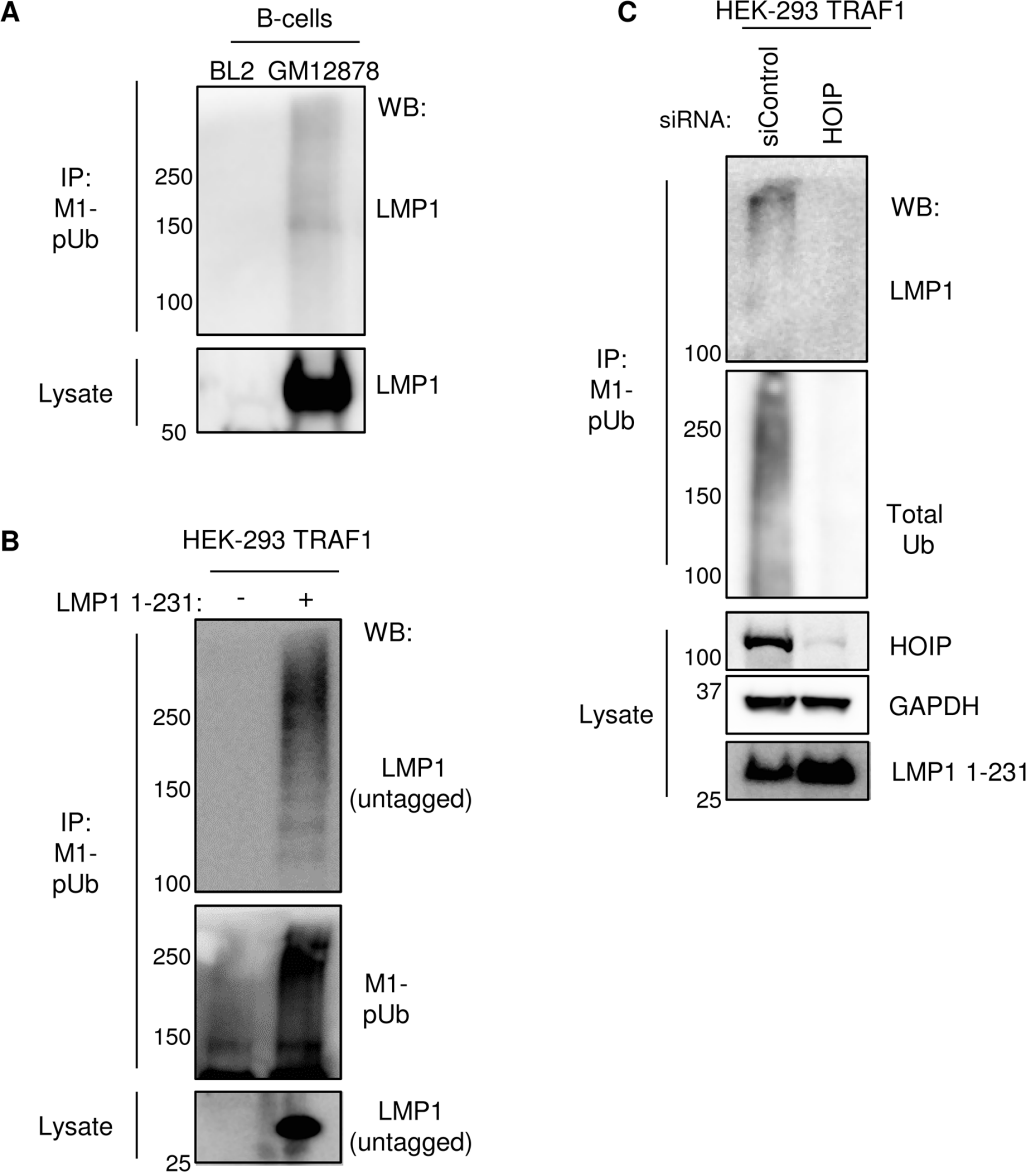
LMP1 is a target of M1-pUb chain attachment. A) M1-pUb chains, immunopurified from control EBV-negative Burkitt lymphoma BL2 cells or from GM12878 LCLs under denaturing conditions, and whole cell lysate were blotted for LMP1. B) M1-pUb chains were immune-purified under denaturing conditions from 293 TRAF1 cells, uninduced or induced for LMP1 1–231 expression for 16 hours. LMP1 1–231 is untagged and has no lysine residues. M1-pUb IPs or whole cell lysate was WB for LMP1 or M1-pUb, as indicated. C) 293 TRAF1 cells were transfected with control or anti-HOIP siRNAs, and 72 hours later, LMP1 1–231 expression was induced for 16 hours. M1-pUb IPs were blotted for LMP1 or total poly-Ub, and lysates were blotted as indicated. A-C are representative of three independent experiments.

The LMP1 N-terminus can be modified by ubiquitin conjugates in transient overexpression assays [85]. We therefore tested whether LMP1 itself might be modified by M1-pUb chain attachment in GM12878 LCLs. EBV-negative BL2 Burkitt lymphoma cells were used as a B-cell negative control. To disrupt protein-protein complexes, GM12878 and BL2 cells were lysed under highly denaturing conditions, using buffer containing 8M urea. M1-pUb chains were then immune-purified, using a M1-pUb-specific monoclonal antibody under denaturing conditions in a buffer that contained 7M urea (Methods) [81]. Immuno-purified M1-pUb material was analyzed by western blot, using an LMP1-specific monoclonal antibody (LMP1 is not epitope tagged in GM12878). Surprisingly, high molecular weight LMP1 conjugates were evident, consistent with the possibility that LMP1 is a direct target of M1-pUb chain attachment (**Fig 7A**). Since WT LMP1 has only a single lysine residue, K330, we hypothesized that it could be the M1-pUb attachment site. We therefore tested whether the lysine-less and untagged LMP1 1–231 molecule was modified by M1-pUb chains in conditional 293 TRAF1 cells. M1-pUb chains were immuno-purified under denaturing conditions from uninduced 293 TRAF1 cells, and from 293 TRAF1 cells induced for LMP1 1–231 expression for 16 hours. M1-pUb immuno-purified material was analyzed by western blot, using an anti-LMP1 monoclonal antibody. Surprisingly, high molecular weight LMP1 conjugates were again identified in extracts from LMP1 1–231+ cells (**Fig 7B**). Notably, high molecular weight LMP1 1–231 species were not observed in M1-pUb pulldowns from 293 TRAF1 cells treated with HOIP siRNAs 72 hours prior to LMP1 1–231 induction, and then western blotted either for M1-pUb or total Ub (**Fig 7C**). These results suggest that LMP1 may be a direct LUBAC target, and support the specificity of the anti-M1-pUb monoclonal antibody. Further studies are required to identify the M1-pUb attachment site in the lysine-less LMP1 1–231 molecule. Of note, all SDS/PAGE samples were boiled in loading buffer with fresh 5% beta-mercaptoethanol, which disrupts cysteine-ubiquitin linkages [86]. Possible LMP1 M1-pUb attachment sites include the LMP1 N-terminus itself or a non-lysine LMP1 residue.

We next used ubiquitination assays to determine whether LUBAC ubiquitinates recombinant TRAF1 *in vitro*. Recombinant N-terminally GST-tagged TRAF1 was added to reaction buffer containing ubiquitin, ATP, the ubiquitin E1 enzyme, as well as the indicated combinations of the ubiquitin E2 enzyme UBE2L3 and LUBAC components (**S10 Fig and Methods**). Reactions were stopped after two hours, boiled in Laemmli sample buffer, and were analyzed by western blot for M1-pUb chain linkages. Interestingly, high-molecular weight, immune-reactive species were abundantly present in lanes 7 and 8, from reactions that contained E1, E2, HOIP, HOIL-1L and TRAF1. The lane 8 reaction also contained SHARPIN. Since SHARPIN was found to be important for M1-pUb attachment to TRAF1 complexes in 293 cells, perhaps protein concentrations used in the *in vitro* Ub assay circumvent the need for SHARPIN’s regulatory role.

### TRAF1 and HOIP are important for LCL growth and survival

**Fig 8:**
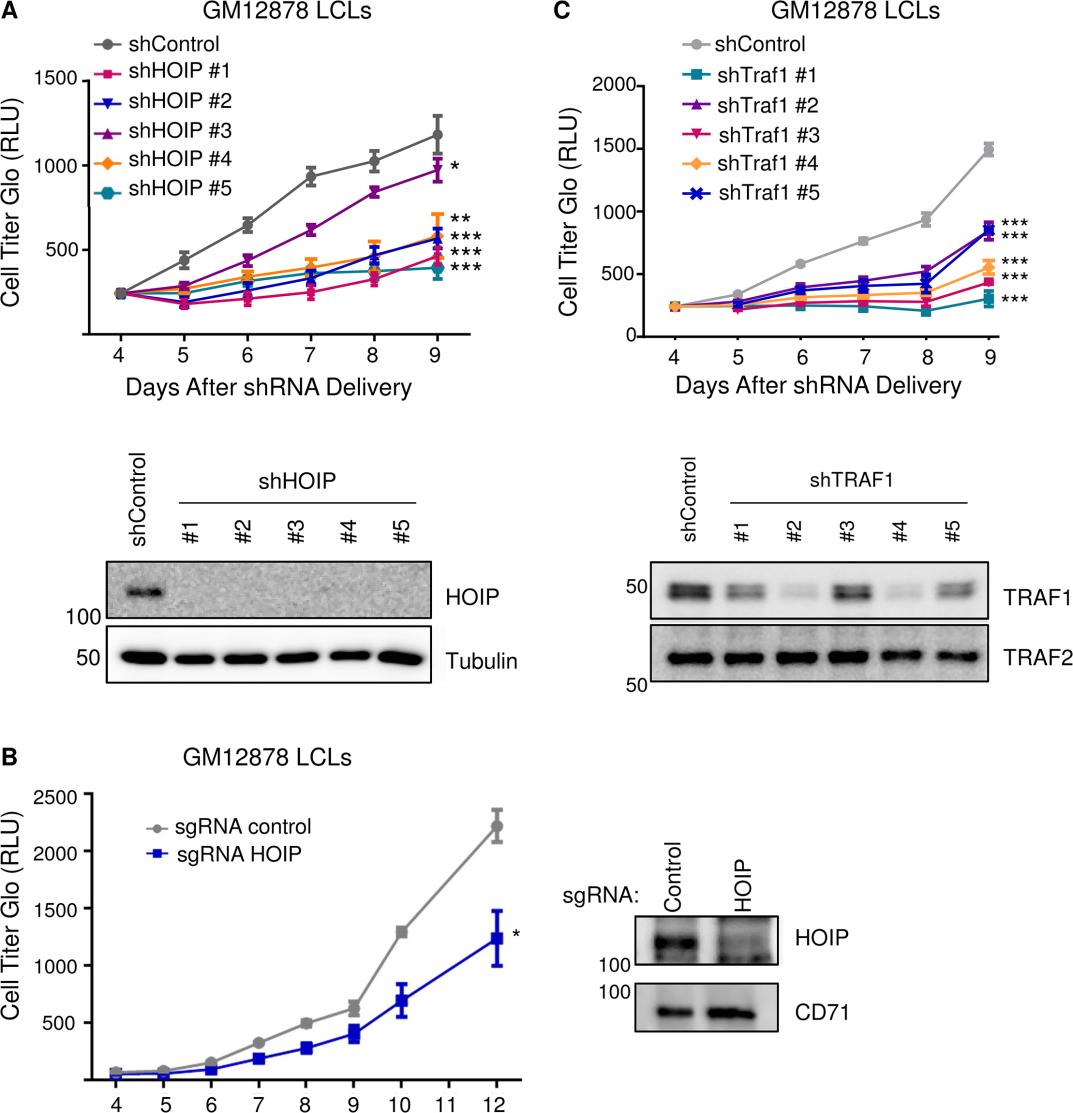
Depletion of HOIP or TRAF1 impairs LCL growth and survival. A) GM12878 LCLs were transduced with lentiviruses that express control shGFP or one of five independent anti-HOIP shRNAs on day 0. Transduced cells were selected with puromcyin on day 2 post-transduction, and then analyzed by quantitative CellTiter-Glo luminescent cell viability assays on the indicated days post transduction. Average and standard deviations from triplicate experiments are shown. WB whole cell lysates obtained four days after transduction are shown below the growth curves. B) GM12878 stable Cas9+ cells were transduced with lentiviruses that express a control anti-GFP sgRNA or an anti-HOIP sgRNA on day 0. Transduced cells were selected by puromcyin on day 2 post-transduction, and then analyzed by CellTiter-glo at the indicated timepoints post-transduction. Western blot of whole cell lysates from Day 6 post-transduction demonstrated HOIP depletion from the cell population. C) GM12878 LCLs were transduced with lentiviruses that express shGFP or one of five independent TRAF1 shRNAs. Transduced cells were selected with puromcyin on day 2, and then analyzed by CellTiter-Glo on the indicated days post-transduction. Average and standard deviations from triplicate experiments are shown. WB of day 4 whole cell lysates are shown below. Student’s one-tailed T-test *P < 0.05, ** P < 0.01, *** P<0.001.

Aberrant HOIP activity has recently been implicated in the pathogenesis of the activated B cell-like (ABC) subtype of diffuse large B-cell lymphoma (DLBCL) [87,88]. LUBAC inhibition was synthetically lethal to ABC DLBCL, but not the germinal center lymphoma subtype, which have lower NF-kB activity [88]. Given these and our results, we tested the effect of HOIP knockdown on GM12878 LCL growth and survival. Interestingly, by comparison with a non-targeting shGFP control, HOIP depletion by five independent shRNAs significantly impaired LCL growth in biological triplicate assays (**Fig 8A**). While four anti-HOIP shRNAs yielded very similar effects, a fifth anti-HOIP shRNA (shRNA #3 on the growth curve) had a statistically significant effect in the same direction, but a more modest growth phenotype. This attenuated phenotype may reflect partial rescue by an off-target shRNA effect, or partial rescue by an alternatively spliced HOIP transcript that lacks the shRNA targeting sequence, and which results in a truncated protein not recognized by our anti-HOIP antibody. To further validate the overall shRNA result, we used CRISPR/Cas9 mutagenesis in GM12878 cells that stably express Cas9 to deplete HOIP (Methods). An anti-HOIP exon 1 small guide RNA (sgRNA) knocked down HOIP expression and caused a statistically significant decrease in LCL growth, by comparison with a control anti-GFP sgRNA (**Fig 8B**). CRISPR/Cas9 edited cells with residual HOIP expression, for example as a result of mono-allelic HOIP disruption, may account for residual LCL growth observed in this experiment. Anti-HOIP sgRNA expression triggered marked induction of caspase 3 and 7 activity, and to a lesser extent, caspase 8 activity **(S11 Fig)**. Western blot analysis of GM12878 whole cell lysates obtained 6 days after transduction with sgRNA-expressing lentiviruses also demonstrated cleaved caspases 3, 7, 9 and cleaved PARP. Overall, these results suggest that HOIP depletion predominantly triggers the intrinsic apoptosis pathway. While TRAF1-independent HOIP roles may also have contributed to this phenotype, it nonetheless suggests an important role for M1-pUb chains in LCL growth and survival, and highlights LUBAC as a potential therapeutic target in EBV-associated lymphoproliferative disorders. We next tested the effect of TRAF1 knockdown in GM12878 LCLs, and found that five independent TRAF1 shRNAs each significantly impaired LCL growth relative to the shRNA control (**Fig 8C**). We note that all five TRAF1 shRNAs had similar effects on LCL growth, despite variation in the extent of TRAF1 knockdown evident on whole cell extract western blot four days after lentivirus transduction. This result raises the possibility that GM12878 are quite sensitive to TRAF1 depletion, and that even partial TRAF1 depletion impaired cell proliferation. Alternatively, off-target shRNA effects may also have contributed to shRNA effects on LCL growth, in particular for shRNA #3, which depleted TRAF1 to the least extent. TRAF1 levels may also have become more similar across shRNA conditions at subsequent timepoints. Nonetheless, the ability of all five TRAF1 shRNAs to impair LCL proliferation argues against off-target effects being solely responsible for the observed phenotypes. Collectively, our results support an important role for TES1 and TRAF1-dependent M1-pUb chains in the LCL immortalized growth phenotype.

### LMP1 and TRAF1 complexes are modified by K63-linked pUb chains

K63-linked pUb chains have important roles in canonical NF-kB and MAP kinase activation pathways. Notably, our proteomic analysis suggested that TRAF1 associated with multiple E3 Ub ligases that catalyze K63-linked pUb chains, including TRAF2, TRAF3, cIAP1, and cIAP2 (**S1 Table**). Likewise, A20, ABIN1 and SQSTM1, each of which have domains that bind to K63-pUb chains, associated with TRAF1 in LMP1 1–231 induced cells. Given also the important role that K63-linked pUb chains play in LUBAC recruitment to TNFR1, we examined whether LMP1 or TRAF1 complexes were decorated by K63-pUb chains. First, we used 293 TRAF1 cells to test whether LMP1 1–231 expression induces attachment of K63-pUb-linked chains to TRAF1 complexes. FLAG-TRAF1 complexes were immuno-purified from 293 TRAF1 cells uninduced or induced for LMP1 1–231 expression for 16 hours, and subjected to western blot analysis with a K63-pUb chain specific antibody [89]. While TRAF1 was not associated with K63-pUb chains in unstimulated 293 cells, TRAF1 complexes were highly modified by K63-pUb chains in extracts from cells that co-express LMP1 1–231 (**S12 Fig**).

**Fig 9:**
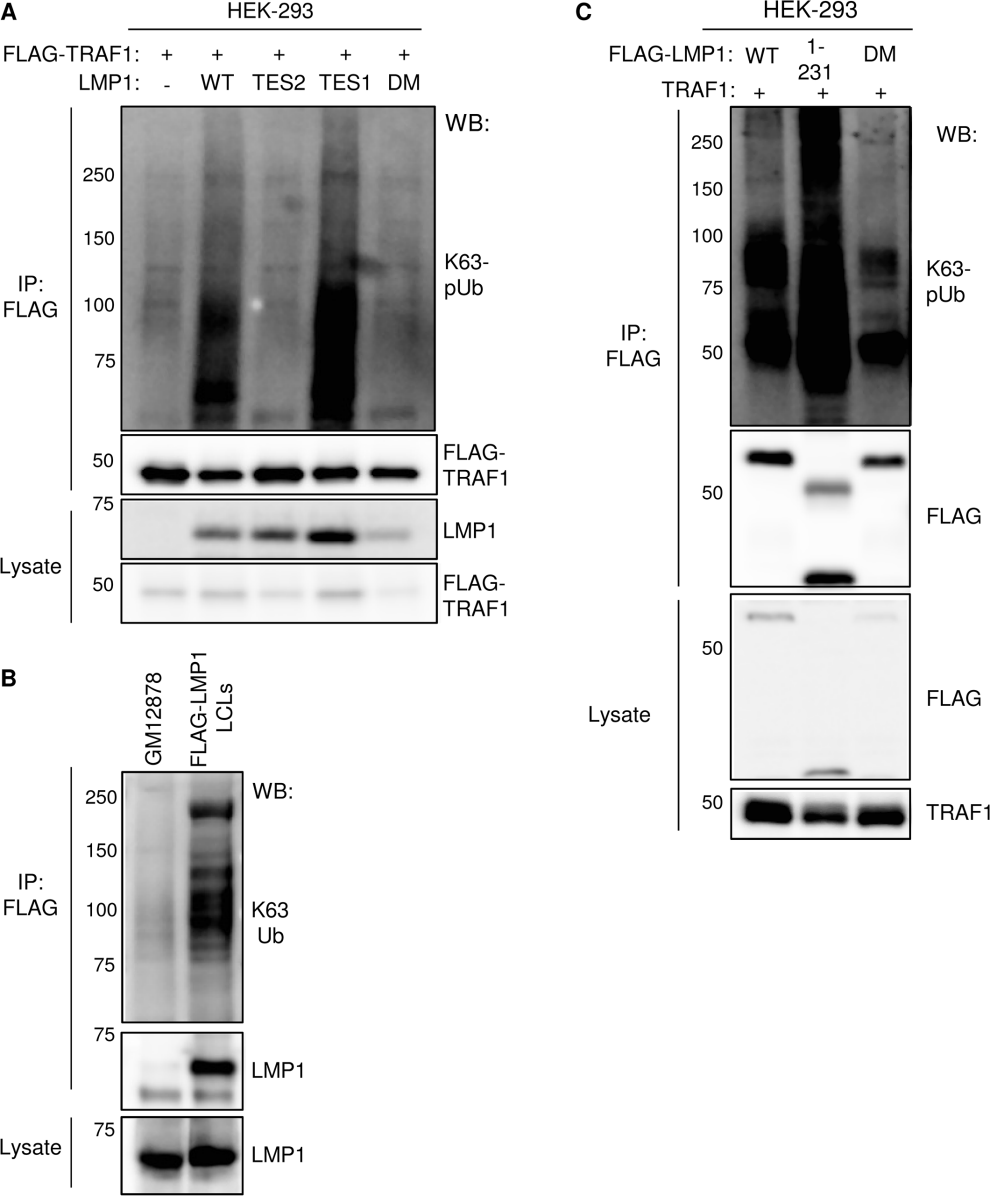
LMP1 TES1 signaling is important for K63-linked pUb chain attachment to TRAF1 and LMP1 complexes. A) 293 TRAF1 cells were co-transfected with empty pSG5 vector (-) or the indicated LMP1 construct, and with FLAG-TRAF1. FLAG-TRAF1 IPs were immunoblotted for K63-pUb, using a K63-pUb chain-specific monoclonal antibody, or for FLAG, as indicated. Lysates were blotted, as indicated. B) FLAG-IPs from GM12878 or FLAG-LMP1 LCLs were blotted for K63-pUb chains or LMP1, as indicated. Lysates were blotted for LMP1. C) FLAG-tagged LMP1 WT, 1–231 or DM constructs were co-transfected with untagged-TRAF1 vector into 293 cells. FLAG-LMP1 immuno-purified complexes or whole cell lysates were blotted, as indicated.

The LMP1 TES2 domain highly activates TRAF6 K63-pUb ligase activity, but does not associate with TRAF1. To test whether LMP1 TES2 signaling nonetheless stimulates K63-pUb attachment to TRAF1 complexes, we co-transfected 293 cells with FLAG-TRAF1 and either WT LMP1, or LMP1 mutants deficient for TES1 and/or TES2 signaling. FLAG-TRAF1 complexes purified from cells that co-expressed wildtype LMP1, or the TES2 null LMP1 mutant, were modified by K63-pUb chains. By contrast, the LMP1 TES1 domain _204_AQAAA_208_ triple point mutant did not stimulate attachment of k63-pUb chains to TRAF1 complexes (**Fig 9A)**. This result is consistent with a model in which TRAF1 recruitment to LMP1 TES1 is important for subsequent K63-pUb attachment to TRAF1 complexes.

We examined whether LMP1 complexes are also modified by K63-pUb chains. Indeed, FLAG-LMP1 complexes, immuno-purified from FLAG-LMP1 LCLs, were modified by K63-pUb chains (**Fig 9B**). To investigate whether LMP1 1–231 signaling is important for K63-pUb chain attachment to LMP1 complexes, 293 cells were co-transfected with FLAG-tagged LMP1 WT, 1–231, or DM vectors and untagged TRAF1. 24 hours after transfection, FLAG-LMP1 immuno-purified complexes were analyzed by western blot for K63-pUb chain attachment (**Fig 9C**). K63-pUb chains decorated WT and 1–231 LMP1 complexes, but not FLAG-LMP1 DM complexes, suggesting that TES1 signaling is important for K63-pUb chain attachment to LMP1 complexes. To identify target(s) of LMP1 TES1-induced K63-pUb chains, we analyzed the K63-pUb chain status of proteins known to associate with the LMP1 TES1 domain. 293 cells were co-transfected with untagged TRAF1, HA-tagged WT LMP1, and FLAG-tagged TRAF1, TRAF2, TRAF3 or GFP negative control, as indicated.

**Fig 10:**
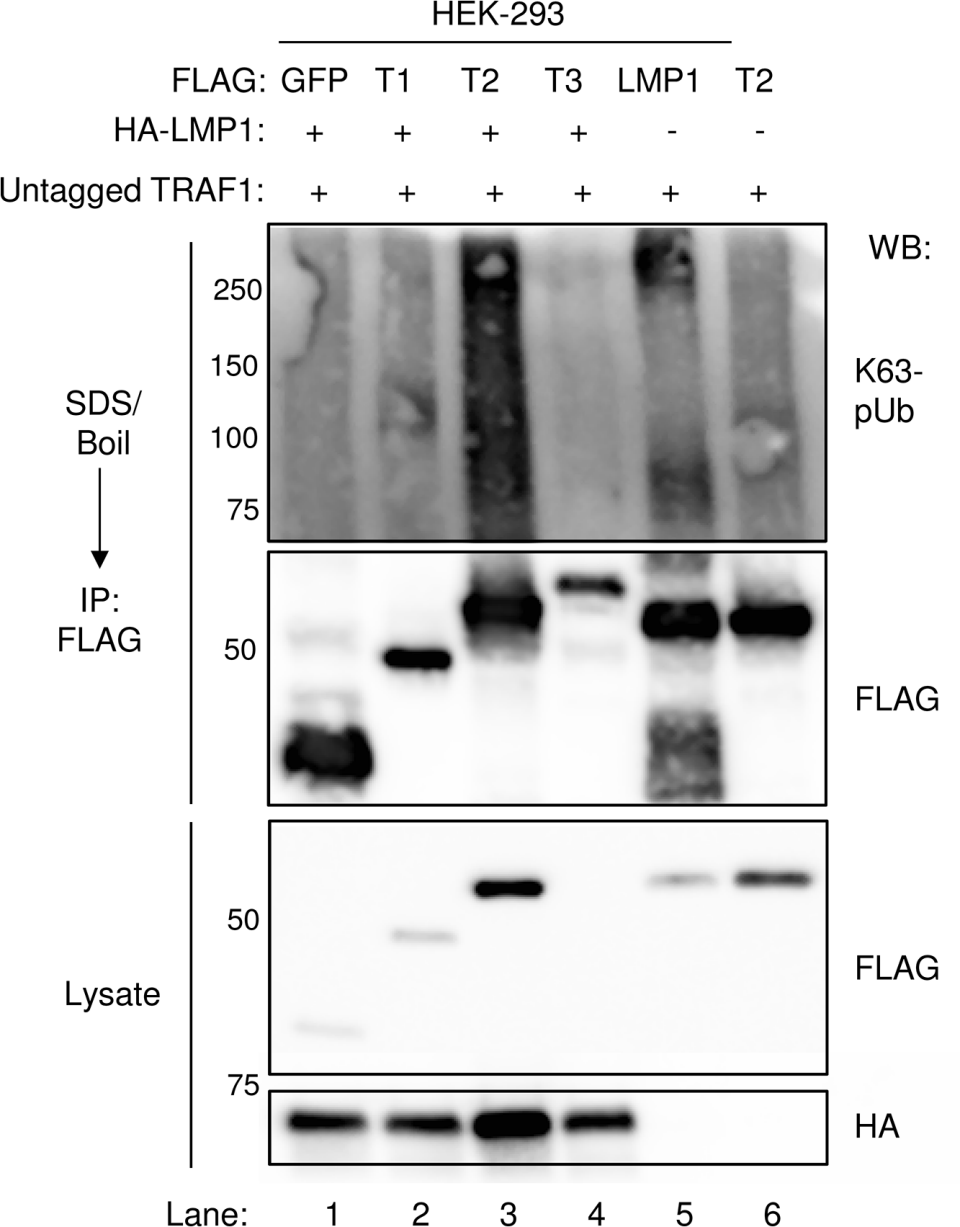
LMP1 1–231 expression induces K63-pUb chain attachment to TRAF2. 293 cells were co-transfected with FLAG-tagged GFP, TRAF1 (T1), TRAF2 (T2), TRAF3 (T3), or LMP1 constructs, HA-LMP1, and untagged TRAF1 for 24 hours, as indicated. 1% SDS was added to whole cell lysates, and samples were boiled for 5 minutes to denature complexes. SDS was diluted to 0.1%, and anti-FLAG IP was performed. Western blots were performed, as indicated. A-D are representative of three independent experiments.

To determine whether LMP1 might itself be modified by K63-linked pUb chains, 293 cells were also co-transfected with untagged TRAF1 and FLAG-LMP1. To denature protein-protein complexes, 1% SDS was added to 293 cell lysates, and samples were boiled for 5 minutes. The SDS concentration was then reduced to 0.1% by addition of NP40 lysis buffer, and FLAG-tagged proteins were immune-purified. Interestingly, western blot analysis demonstrated K63-pUb chain modification of FLAG-TRAF2 (lane 3), whereas signals in other lanes were similar to the FLAG GFP negative control (**Fig 10**). To determine whether LMP1 signaling induced K63-pUb chain attachment on TRAF2, we also analyzed 293 cells co-transfected with TRAF2 and untagged TRAF1, but no LMP1 (lane 6). Interestingly, FLAG-TRAF2 complexes had only background levels of K63-pUb chains when not co-expressed with LMP1, despite similar TRAF2 expression levels. Collectively, our results suggest that LMP1 and TRAF1 stimulate K63-pUb attachment to TRAF2, likely in the context of a TRAF1:TRAF2 heterotrimer.

## Discussion

Abundant TRAF1 expression is a hallmark of multiple EBV-associated human malignancies, including Hodgkin lymphoma and post-transplant lymphoproliferative disorder [47,48,49]. TRAF1 is one of the most highly LMP1-induced genes [42,46,67], and is up-regulated early in the course of EBV-mediated primary B-cell transformation, with close correlation to LMP1 expression [90]. TRAF1 promotes Hodgkin disease Reed-Sternberg cell survival [91]. However, the molecular mechanisms that underlie TRAF1 function downstream of LMP1, or downstream of immune receptors more generally, have remained incompletely understood. In particular, how TRAF1 enhances LMP1 TES1 domain-mediated activation of the JNK [92] and NF-kB pathways [29] have remained uncharacterized.

To gain insight into TRAF1 function, we took a proteomic approach, and found that LMP1 1–231 expression induced association between TRAF1 and LUBAC components. Indeed, TRAF1 complexes purified from GM12878 LCLs contained LUBAC components and were modified by M1-pUb chains. Likewise, we found that LMP1 complexes immuno-purified from LCLs were highly decorated by M1-pUb chains, and LMP1 1–231 expression in 293 TRAF1 cells stimulated LUBAC activity, as judged by the appearance of high molecular weight M1-pUb conjugates by western blot analysis in whole cell extracts. Since TRAF1 associates with the LMP1 TES1 PQQAT motif, likely through interactions with conserved TRAF domain residues or as a heterotrimer with TRAF2 [24], and since most TRAF1 is associated with LMP1 in LCLs [29], our results suggest that a complex containing LMP1, TRAF1 and TRAF2 may be the target of M1-pUb chains. Indeed, TRAF2 depletion impaired the LMP1-induced association between TRAF1 and LUBAC, and reduced M1-pUb chain attachment to TRAF1 complexes. M1-linked-pUb chains play essential roles in NF-kB and JNK activation by TNFR1, CD40, IL1R1, NOD2, and TLR signalosomes [56,93,94,95,96,97,98], though to our knowledge, have not previously been implicated in a pathway downstream of a viral oncoprotein.

**Fig 11:**
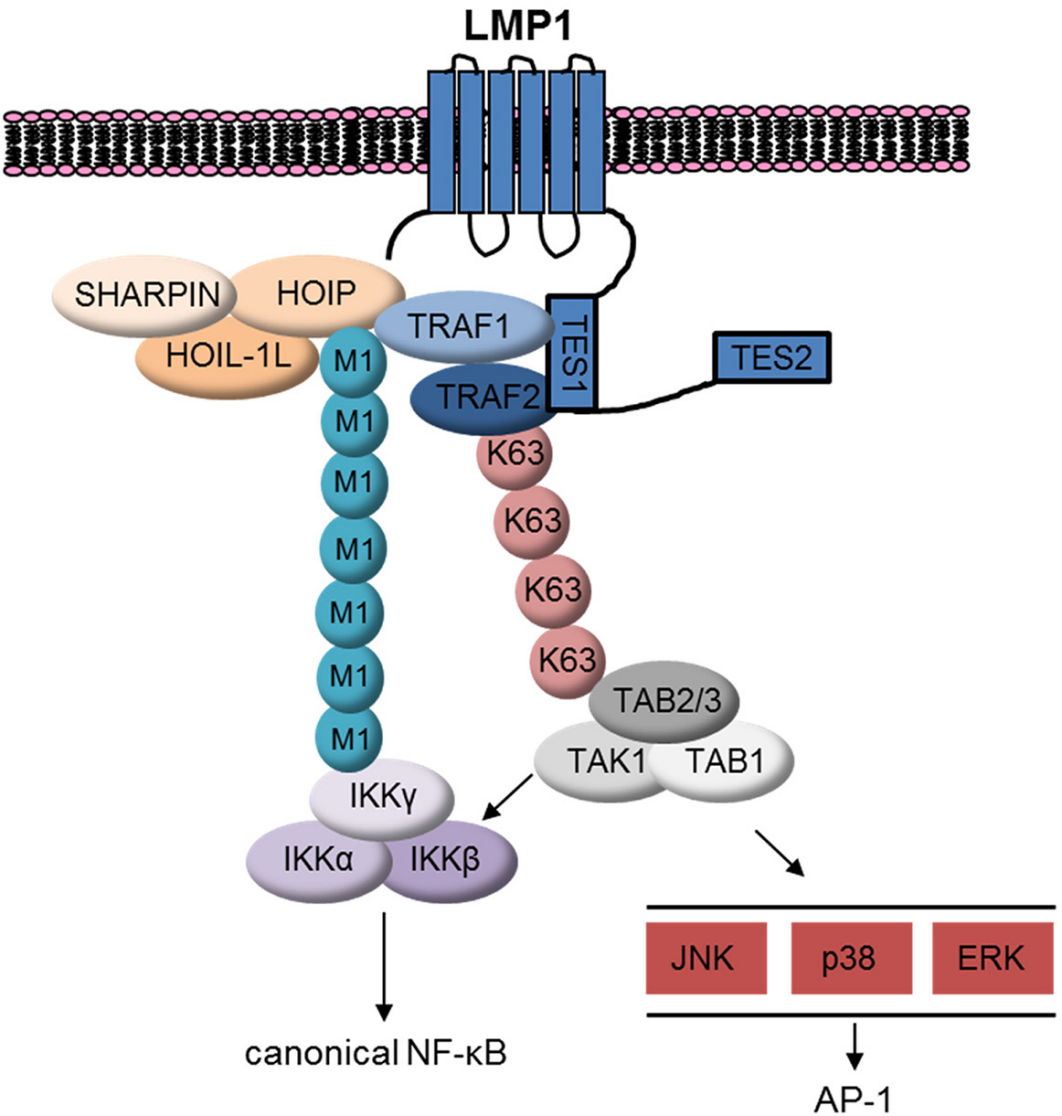
Schematic model. LMP1 recruits TRAF1:TRAF2, which in turn associates with LUBAC. LUBAC attaches M1-pUb chains to LMP1/TRAF1 complexes, which recruits the IKK complex. LMP1 also induces attachment of K63-pUb to TRAF2, which in turn recruits and activates the TAB/TAK1 complex. TAK1 activates downstream MAP kinase and canonical NF-kB pathways.

TRAF1:TRAF2 heterotrimers may also be a target of LMP1 TES1-stimulated K63-pUb chain attachment. LMP1 1–231 induced K63-pUb chain attachment to TRAF2 in 293 TRAF1 cells. K63-pUb chain attachment to TRAF2 may serve important roles in LUBAC recruitment and also in TAK1 activation (**Fig 11**). LUBAC has multiple zinc finger pUb-binding domains, and K63-pUb chains play an important role in LUBAC recruitment to TNFR1 [56,93,94,95]. LMP1-induced K63-pUb chain attachment to TRAF2 may play a similarly important role in LUBAC recruitment to LMP1 and TRAF1 complexes. Likewise, TNF-alpha induced K63-pUb chain attachment to TRAF2 is important for TAB/TAK1 complex recruitment and downstream MAP kinase and canonical NF-kB activation [99–100]. K63-pUb chain linkage to TRAF2 may play a similar role in activating TAK1 downstream of LMP1 TES1. Colocalization of M1- and K63-linked pUb chains may serve to juxtapose the IKK and TAB/TAK1 complexes at the level of LMP1 complexes, and thereby enhance MAP kinase and canonical NF-kB activation (**Fig 11**).

TRAF1:TRAF2 heterotrimers associate with cIAP1 and cIAP2 more tightly than TRAF1 or TRAF2 homotrimers [24]. Since cIAP1/2 catalyzes pUb chains that are essential for LUBAC recruitment to TNFR1, we investigated whether cIAP1/2 were likewise important downstream of LMP1. Surprisingly, cIAP1/2 depletion by SMAC mimetic did not impair LMP1 1-231-induced M1-pUb chain attachment to TRAF1 complexes. While residual cIAP activity could have been sufficient to enable LUBAC recruitment, TNFR1 and LMP1 signaling may differ in this regard. An area of future LMP1 investigation will be to identify the Ub ligase that attaches K63-pUb chains to TRAF2.

We were also intrigued to find that LMP1 may be a target of M1-linked pUb chain attachment in GM12878 LCLs and 293 cells. Western blot analysis of M1-pUb chains, immuno-purified under denaturing conditions from GM12878 or conditional 293 TRAF1 cells, demonstrated high-molecular weight bands reactive with an anti-LMP1 antibody. Since untagged LMP1 1–231 does not have a lysine residue, M1-pUb may therefore be attached to the LMP1 N-terminus or to an LMP1 non-lysine residue. While Kaposi sarcoma associated herpesvirus MIR1 can attach ubiquitin to a MHC class I cysteine residue, all of our western blot samples were treated with reducing agent, which disrupts cysteine-ubiquitin linkages [86]. We note that N-terminally FLAG-tagged LMP1 complexes purified from LCLs are modified by M1-pUb chains. Possible explanations include attachment of M1-pUb chains to FLAG tag lysine residues, to the FLAG N-terminus, to LMP1 K330, or to an LMP1-associated protein, such as TRAF1. Indeed, recombinant TRAF1 was found to be a LUBAC target *in vitro*.

We identified LUBAC as a potential therapeutic target in EBV-transformed B-lymphoblastoid cells. HOIP depletion by independent shRNAs or by CRISPR/Cas9 mutagenesis impaired GM12878 LCL growth and induced apoptosis, largely through activation of the intrinsic apoptosis pathway. Interestingly, LUBAC has recently been implicated in the pathogenesis of the ABC subtype of DLBCL, and a stapled alpha-helical peptide inhibitor that blocks HOIP and HOIL-1L association is toxic to DLBCL [87,88]. A goal of future studies will be to identify whether anti-LUBAC stapled peptides inhibit the growth of EBV-transformed B-cells. Similarly, LMP1 highly upregulates TRAF1 expression in transfected keratinocytes, and TRAF1 expression was detectable in 17 of 42 EBV+ undifferentiated nasopharyngeal carcinomas (NPC) [50]. Further studies are required to determine whether TRAF1 associates with LUBAC in the context of NPC, whether TRAF1 or LMP1 co-localize with M1- or K63-chains in NPC tumor samples, and whether HOIP depletion is toxic to EBV+ NPC cells in culture.

## Supporting Information

S1 Fig HEK-293 TRAF1 cells and GM12878 TRAF1 cells stably express TRAF1 at physiological levels. A. Whole cell lysates from GM12878 LCLs, HEK-293 cells with conditional LMP1 1–231 expression, or HEK-TRAF1 cells with conditional LMP1 1–231 expression were blotted, as indicated. B. Lysates from GM1278 LCLs, or GM12878 with stable FLAG-TRAF1 expression, were blotted for TRAF1. FLAG-tagged TRAF1 is present at similar levels as endogenous TRAF1. Blots are representative of triplicate experiments.
(TIF)

S2 Fig TRAF1 enhances LMP1 TES1-mediated TAK1 activation. Whole cell extracts from 293, or 293 TRAF1 cells, uninduced or induced for LMP1 1–231 expression for 16 hours, as indicated, were blotted for phosphoTAK1, tubulin or LMP1, as indicated. Blots are representative of triplicate experiments.
(TIF)

S3 Fig SAINT analysis identifies high-confidence TRAF1 interactors. FLAG- TRAF1 was affinity purified from 293 TRAF cells that were either uninduced, or induced for LMP1 1–231 expression for 16 hours. FLAG-GFP was affinity purified from 293 FLAG-GFP cells induced for LMP1 1–231 expression for 16 hours. Independent replicates of affinity purified FLAG-TRAF1 or FLAG-GFP control were analyzed by LC-MS/MS. Using 30 additional 293 cell FLAG controls, the SAINT algorithm was then used to identify high-confidence TRAF1 Interacting proteins in each condition. A SAINT score of Avg P ≥ 0.80 has an estimated FDR of ≤1%. See **Methods** for details. HOIP, A20 and HOIL-1L scores are indicated in red.
(TIF)

S4 Fig SHARPIN associates with TRAF1 and LMP1 in GM12878 cells. Control HA-IKK-epsilon or HA-SHARPIN were immuno-purified from GM12878 stable cell lines. HA-IPs were blotted, as indicated. Blots are representative of triplicate experiments.
(TIF)

S5 Fig LMP1 1–231 induces co-localization between TRAF1 and the UBAN-GFP M1-pUb chain sensor. 293 cells were transiently transfected with UBAN-GFP (top panel), or with UBAN-GFP, FLAG-TRAF1 and LMP1 (bottom four panels). Cells were fixed, permeabilized, and immunostained for TRAF1 (cyan) and LMP1 (red), and imaged by confocal microscopy. Image analysis was performed with ImageJ/Fiji software.
(TIF)

S6 Fig TRAF1 and IKK-gamma associate in GM12878 cells. A) FLAG-GFP or FLAG-TRAF1 complexes were Immuno-purified from GM12878 stable cell lines. FLAG-IPs and lysates were blotted, as indicated. B) FLAG-GFP or FLAG-IKK-gamma were immuno-purified from GM12878 stable cell lines. FLAG-IPs and lysates were blotted, as indicated. The artifact present just above HA-IKK-gamma in the HA-GFP GM12878 lysate did not immuno-precipitate. Blots are representative of triplicate experiments.
(TIF)

S7 Fig TRAF2 is important for LUBAC recruitment and M1-pUb chain attachment to TRAF1 complexes. A. 72 hours following 293 TRAF1 cell transfection with the indicated siRNAs, LMP1 1–231 expression was induced for 16 hours. Immuno-purified FLAG-TRAF1 complexes or whole cell lysates were immuno-blotted, as indicated. B. Whole cell lysates from A were immuno-blotted, as indicated. C. 72 hours after transfection with non-targeting siControl or TRAF2 siRNA, LMP1 1–231 expression was induced in 293 TRAF1 cells for 16 hours, and TRAF1 immuno-precipitated complexes or whole cell lysates were blotted, as indicated. Blots are representative of triplicate experiments.
(TIF)

S8 Fig Validation of siRNA depletion of HOIL-1L mRNA. Since tested available antibodies did not detect endogenously expressed HOIL-1L in our HEK-293 cells, HOIL-1L siRNA target knockdown efficiency was validated by real-time PCR in a parallel experiment. 96 hours after 293 cell transfection with a non-targeting siRNA control vs a siRNA against HOIL-1L, RNA was extracted and subjected to qPCR analysis. HOIL-1L mRNA was normalized to an 18S rRNA control to control for cell number. Normalized HOIL-1L levels in non-targeting siRNA control-treated cells were set to 1. Shown are the average and standard deviation of triplicate measurements. *Student’s 1-tailed T-test P<.01.
(TIF)

S9 Fig Effect of LUBAC knockdown on LMP1 1–231 mediated MAP kinase and canonical NF-kB pathway activation in 293 TRAF1 cells. 72 hours after transfection with control siRNA, or siRNAs against HOIP and HOIL-1L, 293 TRAF1 cells were induced for LMP1 1–231 expression overnight. Whole cell lysates were immuno-blotted, as indicated. Blots are representative of triplicate experiments.
(TIF)

S10 Fig LUBAC modifies recombinant GST-TRAF1 in vitro. In vitro ubiquitination assays were performed with the indicated components. Reactions were immuno-blotted for M1-pUb chains. See **Methods** for experimental details. The results are representative of triplicate experiments.
(TIF)

S11 Fig CRISPR/Cas9-mediated HOIP depletion triggers caspase activation and PARP cleavage in GM12878 LCLs. A. Caspase-Glo 3/7 and 8 assays were performed on GM12878 Cas9+ LCLs six days after the introduction of control anti-GFP or anti-HOIP exon 1 sgRNAs. Caspase-Glo 3/7 measures the combined activity of caspases 3 and 7. ***P<.001, *P<.05 (Student’s 1 tailed T-test). B. Western blot analysis of whole cell lysates obtained from GM12878 cells assayed in panel A, and were representative of triplicate experiments.
(TIF)

S12 Fig LMP1 1–231 induces K63-pUb chain attachment to TRAF1 complexes. 293 cells were co-transfected with FLAG-GFP or FLAG-TRAF1, and with pSG5 empty vector control or LMP1 1–231, as indicated. Immuno-purified FLAG complexes and whole cell lysates were blotted, as indicated. Blots are representative of triplicate expreiments.
(TIF)

S1 Table AP/MS datasets and SAINT analysis. Shown on worksheet 1 are the Protein ID, Gene ID, and SAINT scores for uninduced 293 TRAF1 and 293 TRAF1 induced for LMP1 1–231 expression. Also shown are the total spectral counts for TRAF1 and GFP AP/MS analyses. Worksheet two displays the SAINT scores, and the total spectral counts for our AP/MS data and Control datasets used in the analysis.
(XLSX)
