# Supplementary figures and images for: TRAF1 Coordinates Polyubiquitin Signaling to Enhance Epstein-Barr Virus LMP1-Mediated Growth and Survival Pathway Activation
Source: PLoS Pathog. 2015 May 21;11(5):e1004890. doi: 10.1371/journal.ppat.1004890 (PMC4440769; doi:10.1371/journal.ppat.1004890)

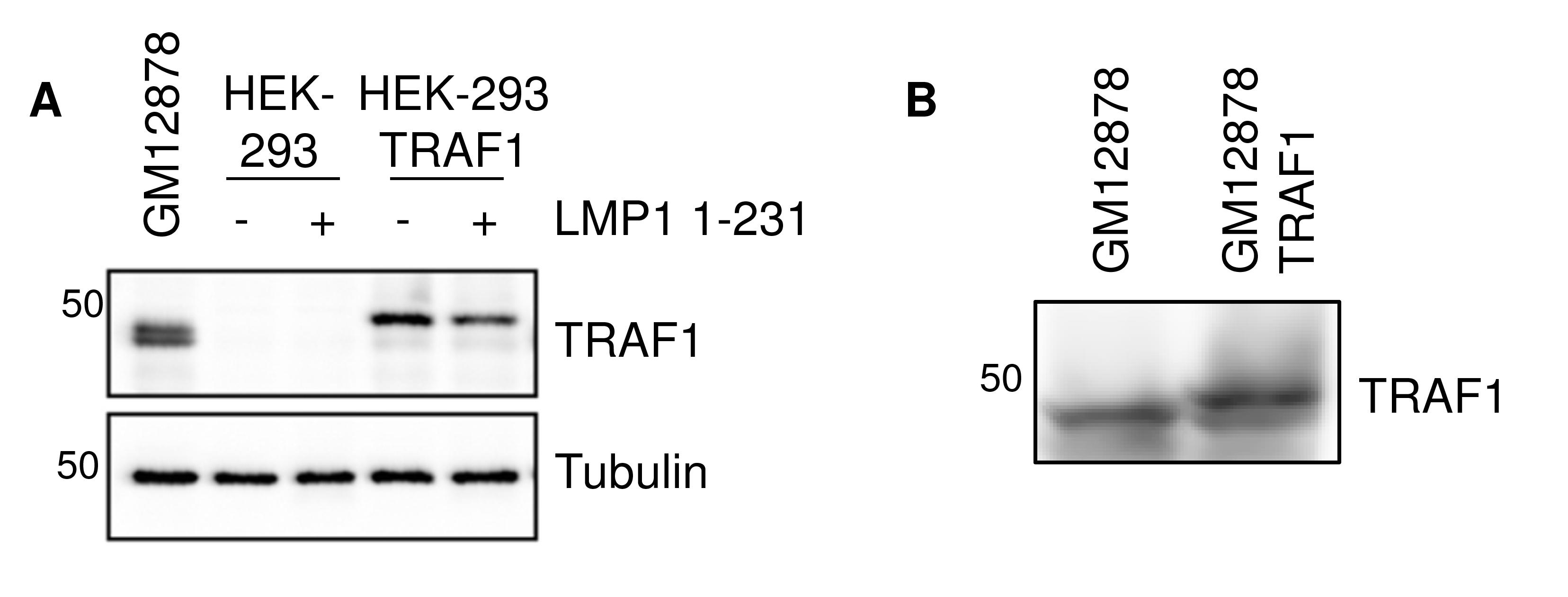

Supplement: S1 Fig — A. Whole cell lysates from GM12878 LCLs, HEK-293 cells with conditional LMP1 1–231 expression, or HEK-TRAF1 cells with conditional LMP1 1–231 expression were blotted, as indicated. B. Lysates from GM1278 LCLs, or GM12878 with stable FLAG-TRAF1 expression, were blotted for TRAF1. FLAG-tagged TRAF1 is present at similar levels as endogenous TRAF1. Blots are representative of triplicate experiments. (TIF) [file ppat.1004890.s001.tif]

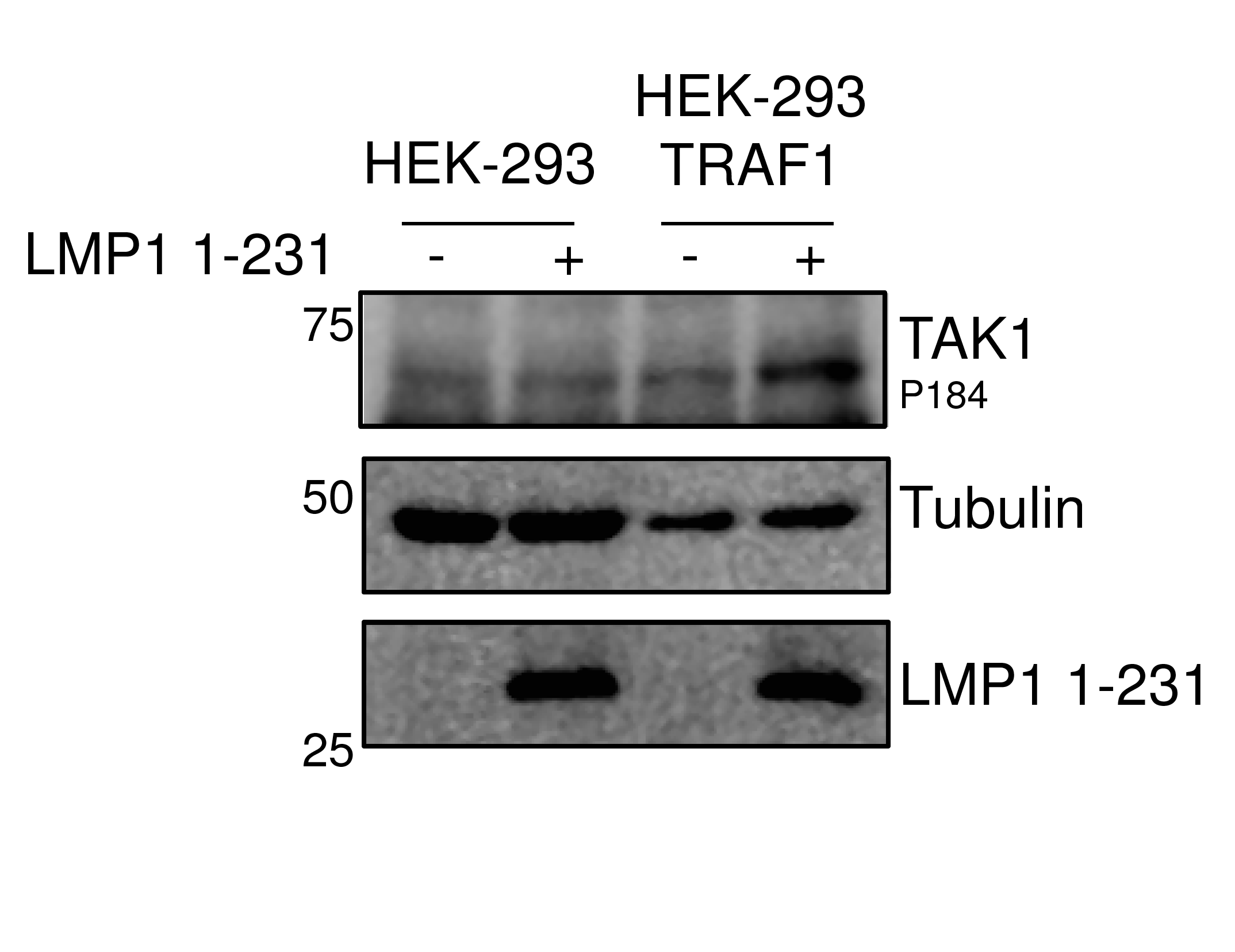

Supplement: S2 Fig — Whole cell extracts from 293, or 293 TRAF1 cells, uninduced or induced for LMP1 1–231 expression for 16 hours, as indicated, were blotted for phosphoTAK1, tubulin or LMP1, as indicated. Blots are representative of triplicate experiments. (TIF) [file ppat.1004890.s002.tif]

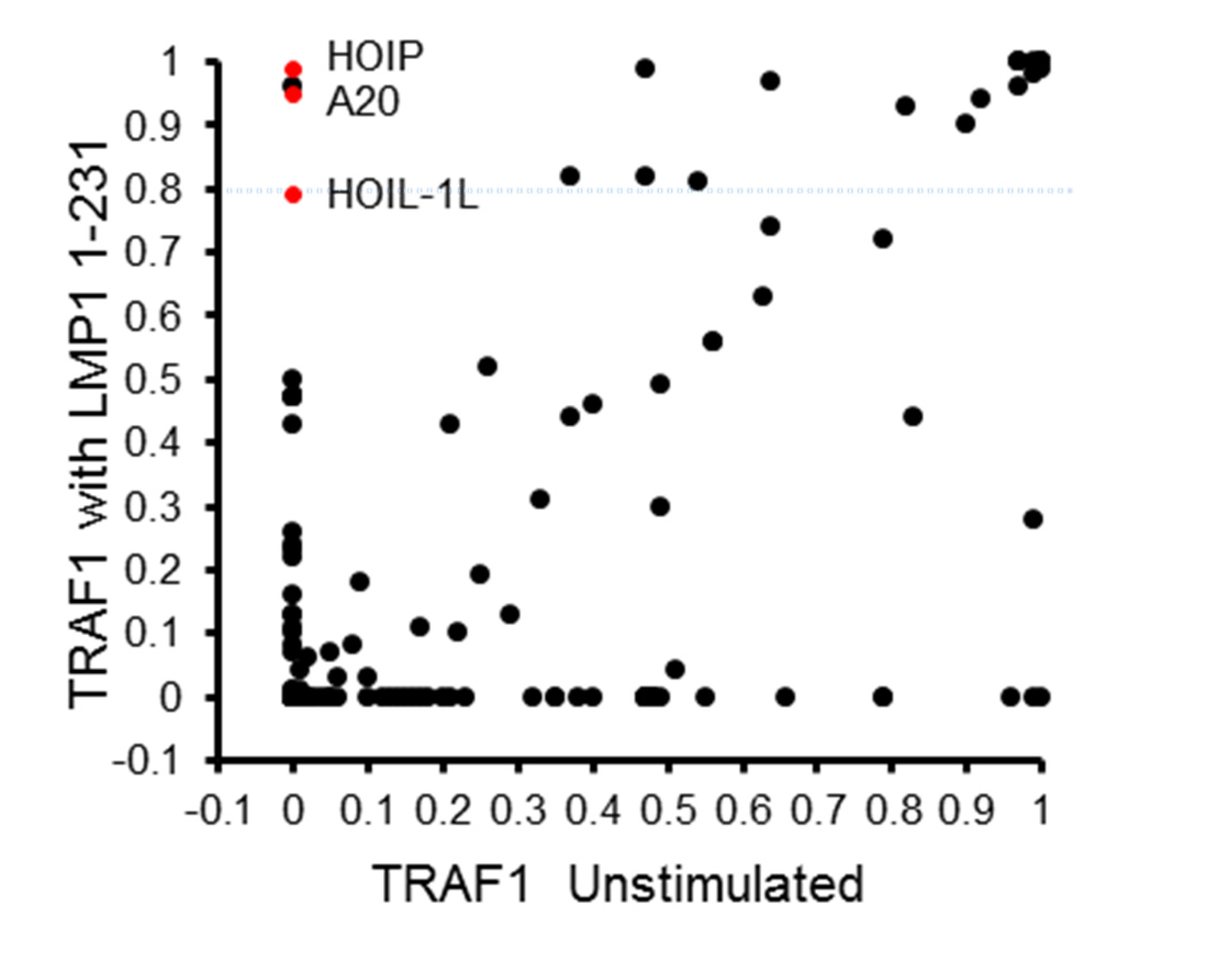

Supplement: S3 Fig — FLAG- TRAF1 was affinity purified from 293 TRAF cells that were either uninduced, or induced for LMP1 1–231 expression for 16 hours. FLAG-GFP was affinity purified from 293 FLAG-GFP cells induced for LMP1 1–231 expression for 16 hours. Independent replicates of affinity purified FLAG-TRAF1 or FLAG-GFP control were analyzed by LC-MS/MS. Using 30 additional 293 cell FLAG controls, the SAINT algorithm was then used to identify high-confidence TRAF1 Interacting proteins in each condition. A SAINT score of Avg P ≥ 0.80 has an estimated FDR of ≤1%. See Methods for details. HOIP, A20 and HOIL-1L scores are indicated in red. (TIF) [file ppat.1004890.s003.tif]

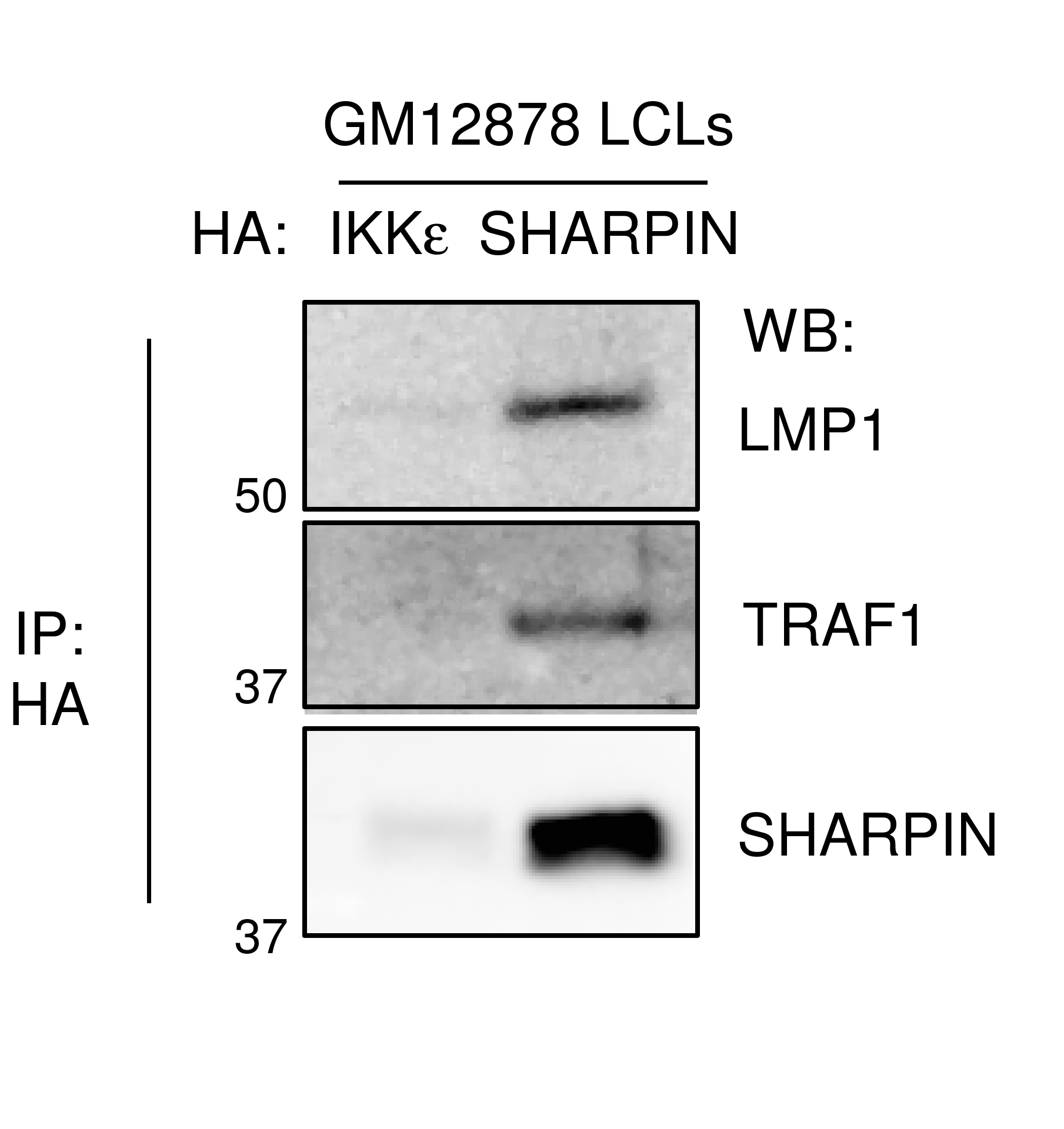

Supplement: S4 Fig — Control HA-IKK-epsilon or HA-SHARPIN were immuno-purified from GM12878 stable cell lines. HA-IPs were blotted, as indicated. Blots are representative of triplicate experiments. (TIF) [file ppat.1004890.s004.tif]

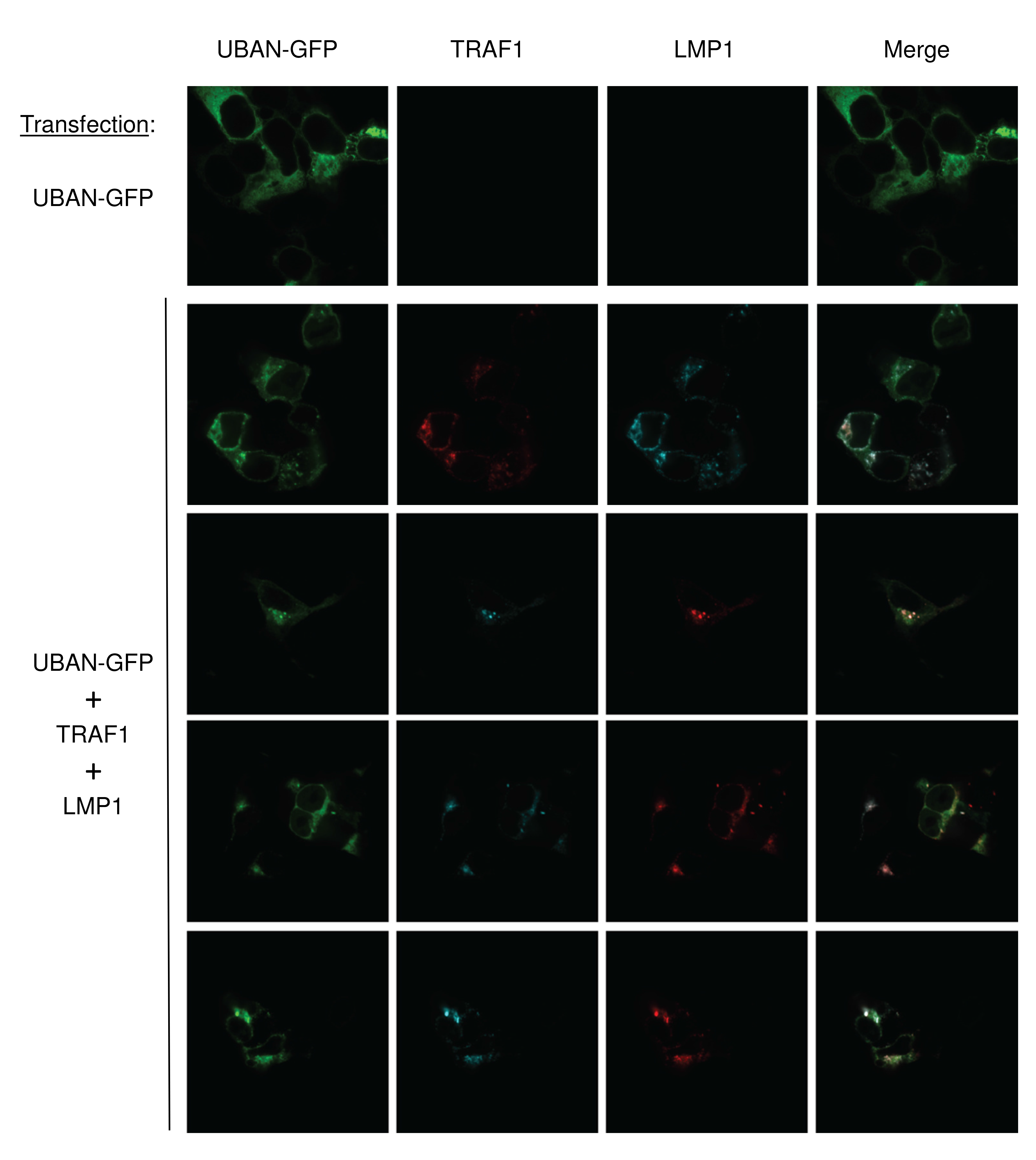

Supplement: S5 Fig — 293 cells were transiently transfected with UBAN-GFP (top panel), or with UBAN-GFP, FLAG-TRAF1 and LMP1 (bottom four panels). Cells were fixed, permeabilized, and immunostained for TRAF1 (cyan) and LMP1 (red), and imaged by confocal microscopy. Image analysis was performed with ImageJ/Fiji software. (TIF) [file ppat.1004890.s005.tif]

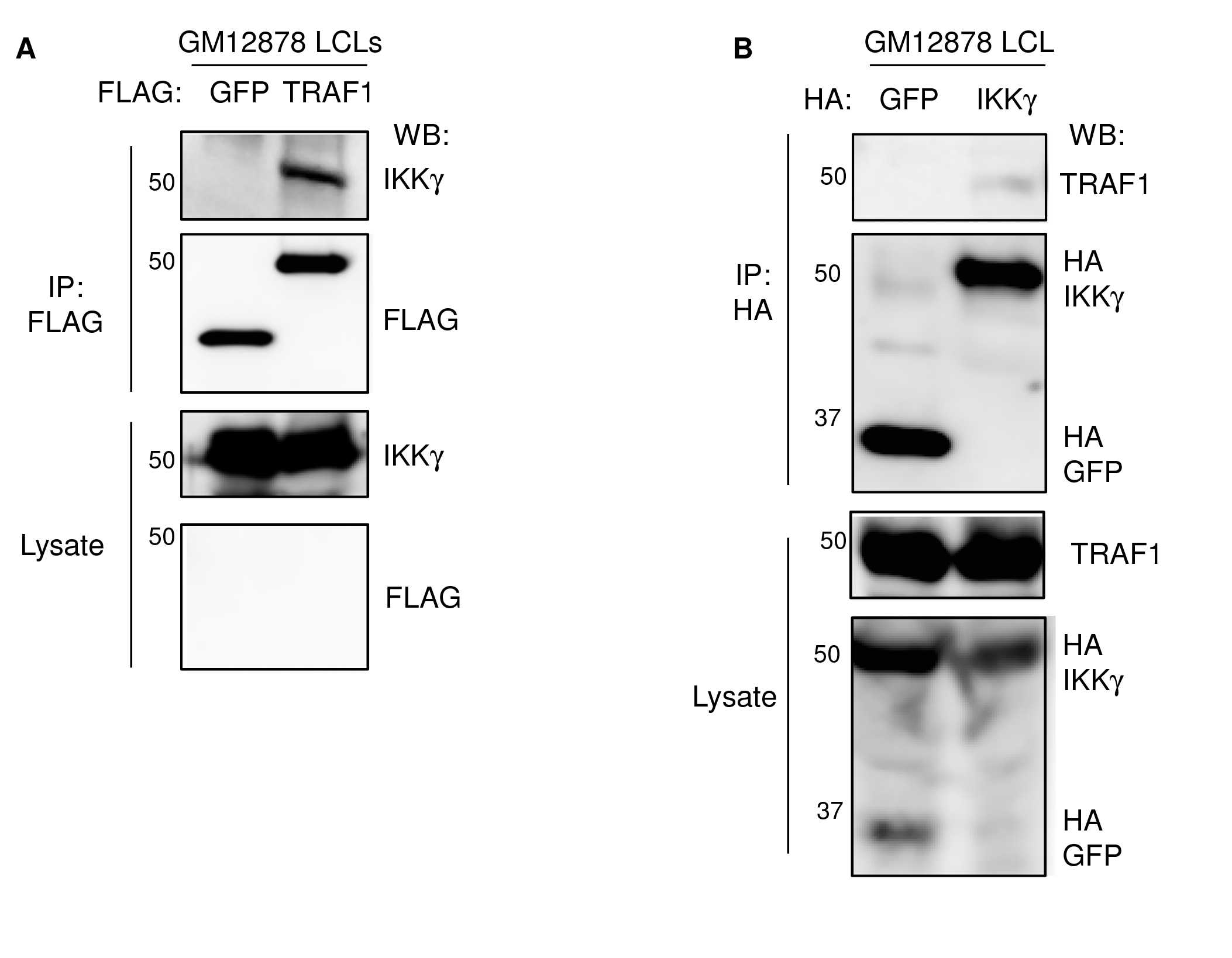

Supplement: S6 Fig — A) FLAG-GFP or FLAG-TRAF1 complexes were Immuno-purified from GM12878 stable cell lines. FLAG-IPs and lysates were blotted, as indicated. B) FLAG-GFP or FLAG-IKK-gamma were immuno-purified from GM12878 stable cell lines. FLAG-IPs and lysates were blotted, as indicated. The artifact present just above HA-IKK-gamma in the HA-GFP GM12878 lysate did not immuno-precipitate. Blots are representative of triplicate experiments. (TIF) [file ppat.1004890.s006.tif]

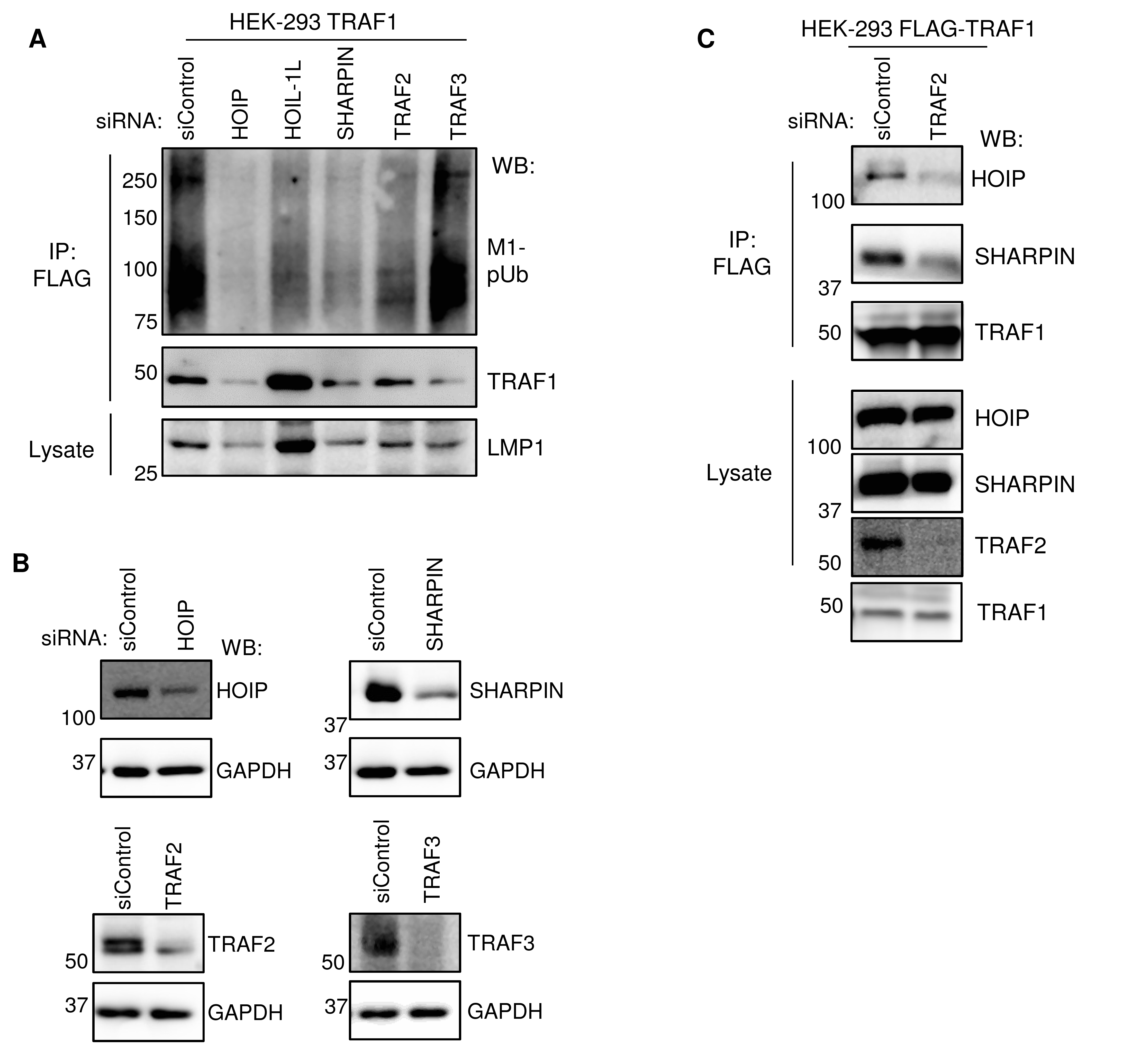

Supplement: S7 Fig — A. 72 hours following 293 TRAF1 cell transfection with the indicated siRNAs, LMP1 1–231 expression was induced for 16 hours. Immuno-purified FLAG-TRAF1 complexes or whole cell lysates were immuno-blotted, as indicated. B. Whole cell lysates from A were immuno-blotted, as indicated. C. 72 hours after transfection with non-targeting siControl or TRAF2 siRNA, LMP1 1–231 expression was induced in 293 TRAF1 cells for 16 hours, and TRAF1 immuno-precipitated complexes or whole cell lysates were blotted, as indicated. Blots are representative of triplicate experiments. (TIF) [file ppat.1004890.s007.tif]

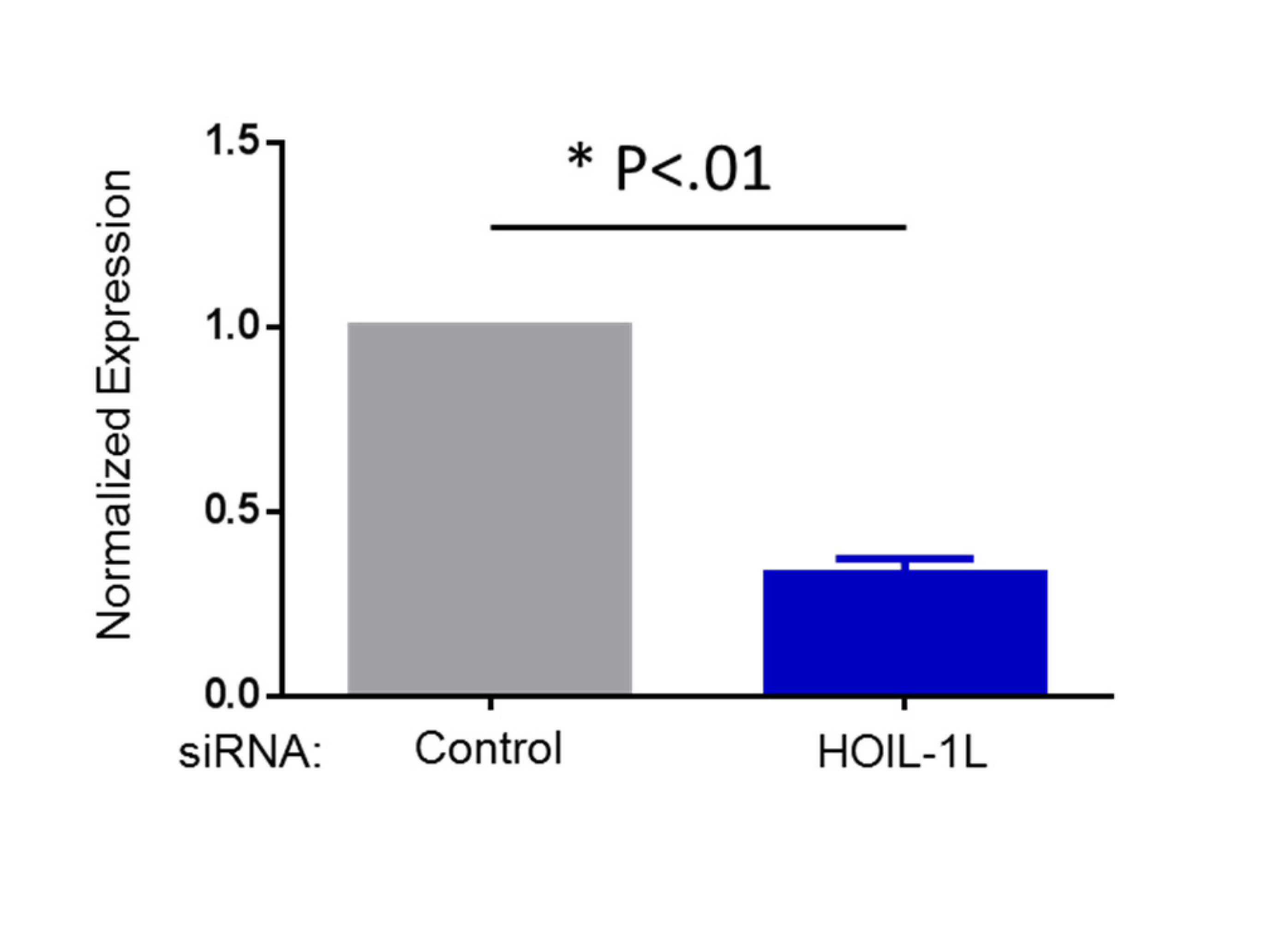

Supplement: S8 Fig — Since tested available antibodies did not detect endogenously expressed HOIL-1L in our HEK-293 cells, HOIL-1L siRNA target knockdown efficiency was validated by real-time PCR in a parallel experiment. 96 hours after 293 cell transfection with a non-targeting siRNA control vs a siRNA against HOIL-1L, RNA was extracted and subjected to qPCR analysis. HOIL-1L mRNA was normalized to an 18S rRNA control to control for cell number. Normalized HOIL-1L levels in non-targeting siRNA control-treated cells were set to 1. Shown are the average and standard deviation of triplicate measurements. *Student’s 1-tailed T-test P<.01. (TIF) [file ppat.1004890.s008.tif]

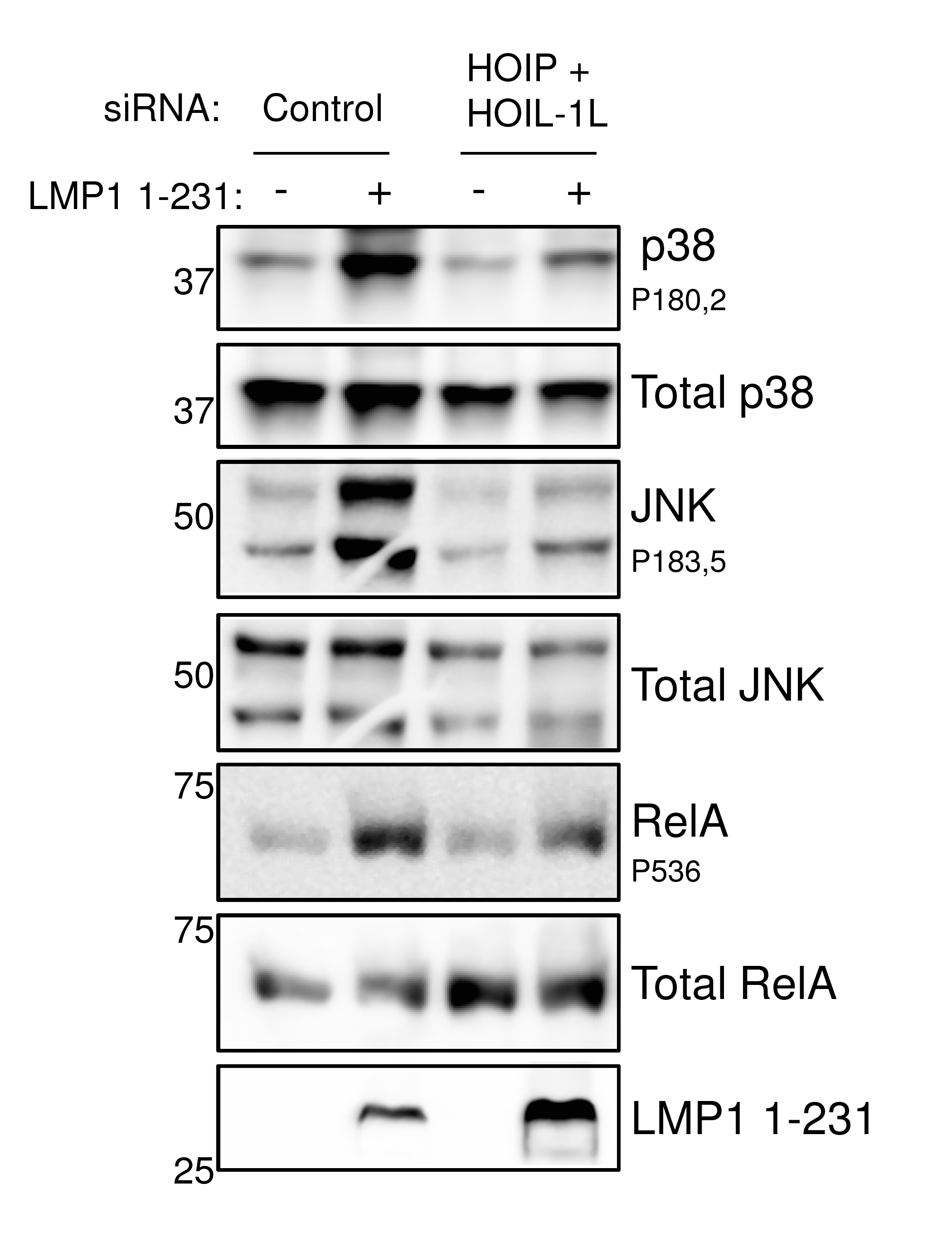

Supplement: S9 Fig — 72 hours after transfection with control siRNA, or siRNAs against HOIP and HOIL-1L, 293 TRAF1 cells were induced for LMP1 1–231 expression overnight. Whole cell lysates were immuno-blotted, as indicated. Blots are representative of triplicate experiments. (TIF) [file ppat.1004890.s009.tif]

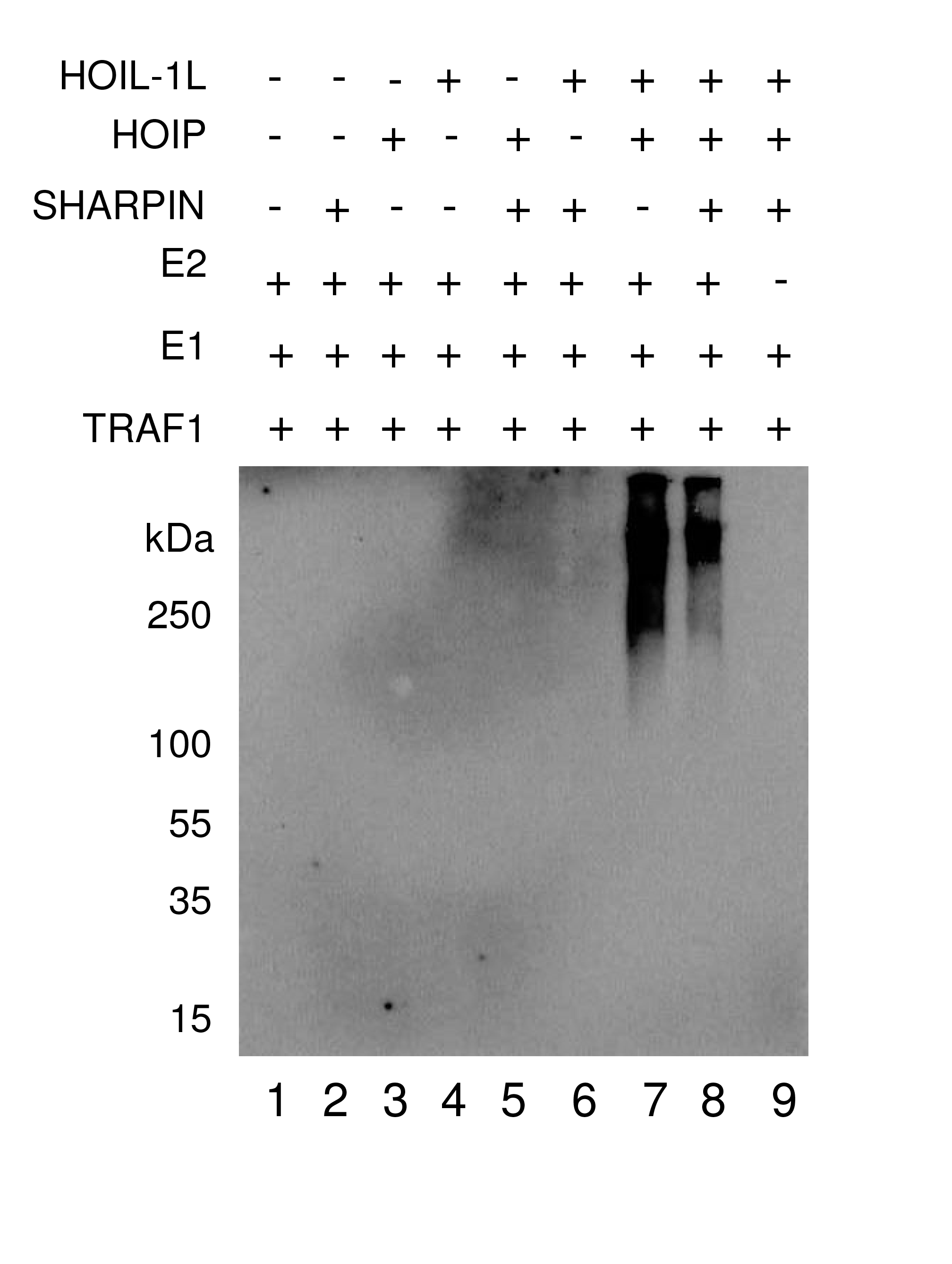

Supplement: S10 Fig — In vitro ubiquitination assays were performed with the indicated components. Reactions were immuno-blotted for M1-pUb chains. See Methods for experimental details. The results are representative of triplicate experiments. (TIF) [file ppat.1004890.s010.tif]

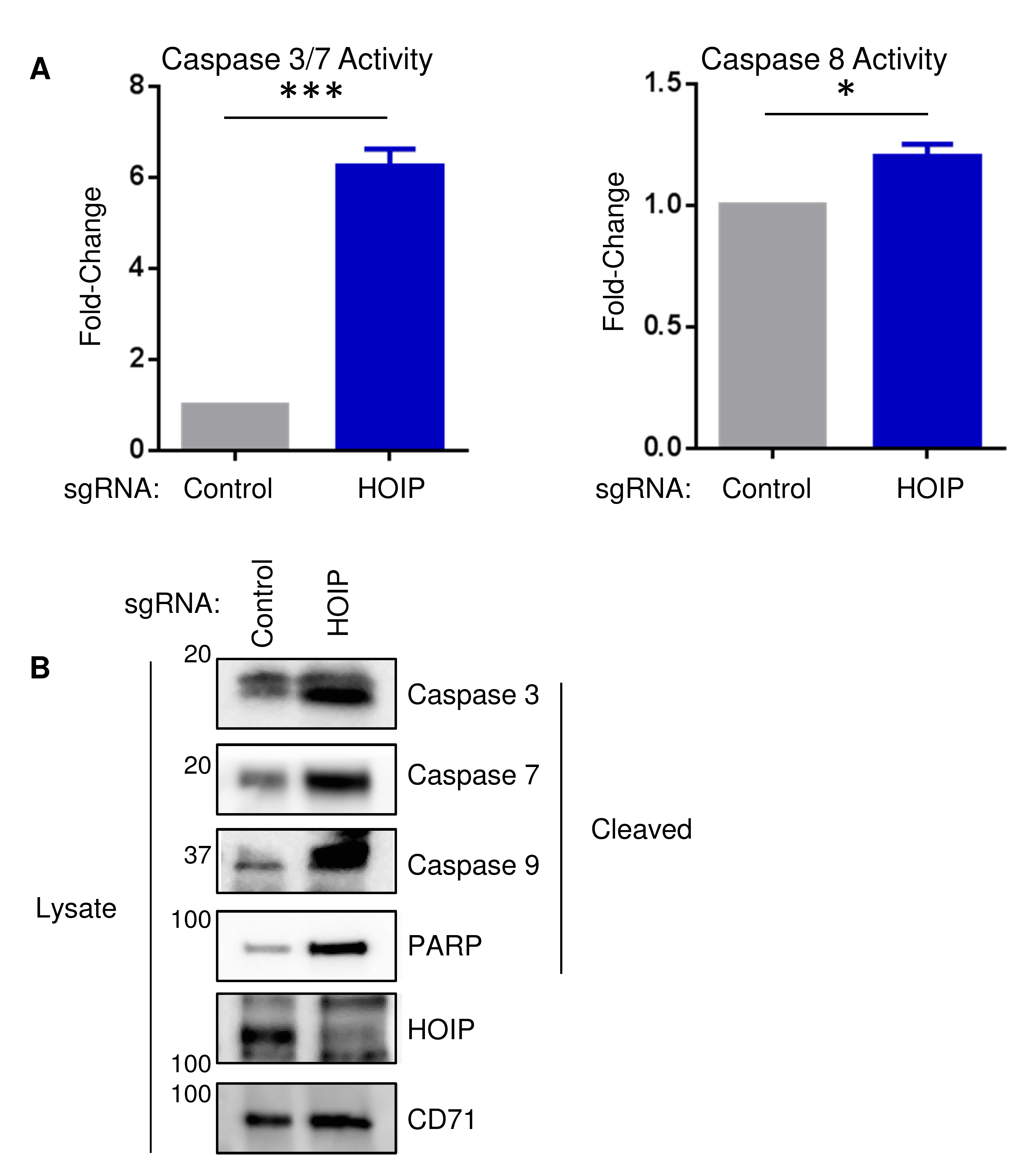

Supplement: S11 Fig — A. Caspase-Glo 3/7 and 8 assays were performed on GM12878 Cas9+ LCLs six days after the introduction of control anti-GFP or anti-HOIP exon 1 sgRNAs. Caspase-Glo 3/7 measures the combined activity of caspases 3 and 7. ***P<.001, *P<.05 (Student’s 1 tailed T-test). B. Western blot analysis of whole cell lysates obtained from GM12878 cells assayed in panel A, and were representative of triplicate experiments. (TIF) [file ppat.1004890.s011.tif]

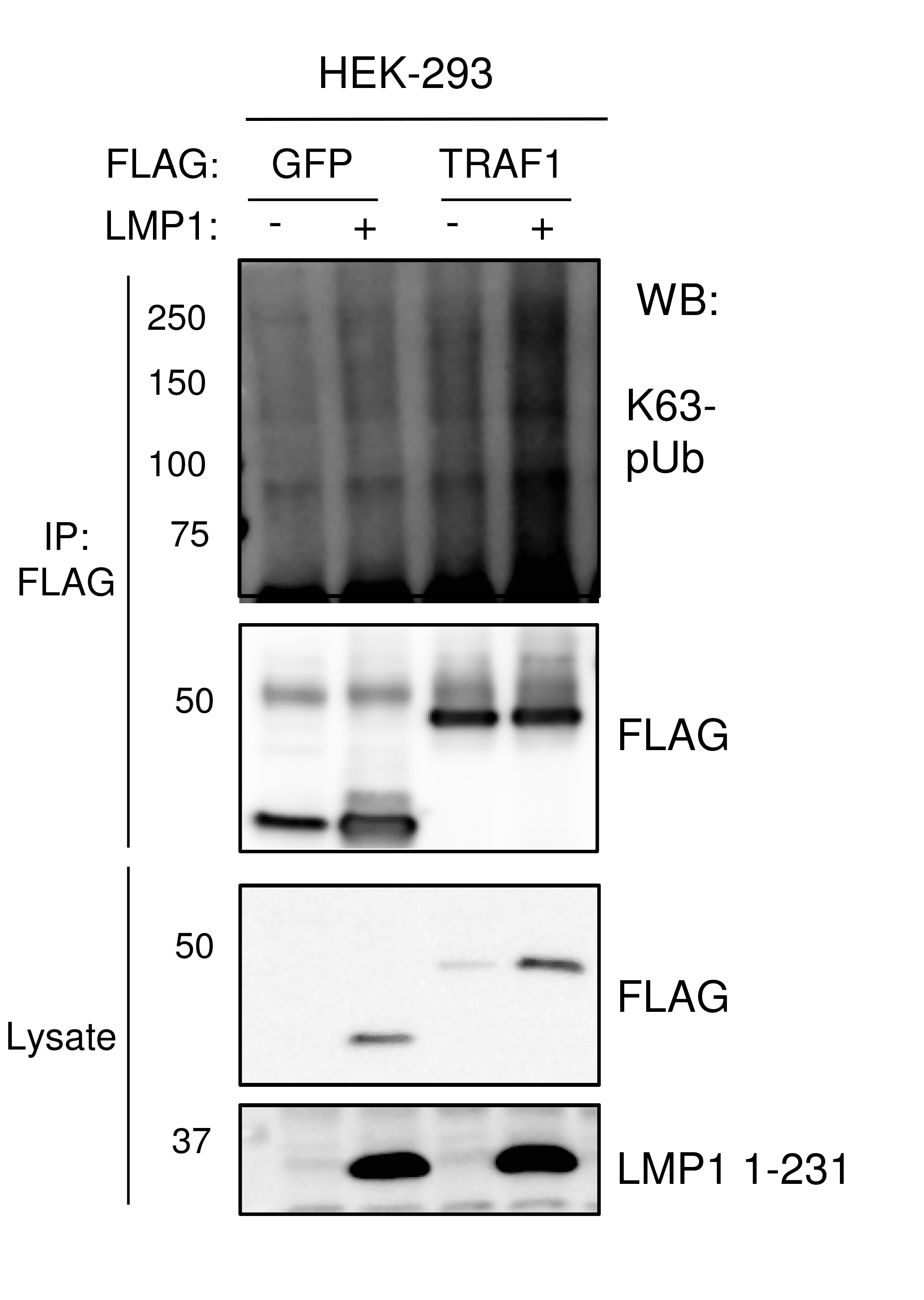

Supplement: S12 Fig — 293 cells were co-transfected with FLAG-GFP or FLAG-TRAF1, and with pSG5 empty vector control or LMP1 1–231, as indicated. Immuno-purified FLAG complexes and whole cell lysates were blotted, as indicated. Blots are representative of triplicate expreiments. (TIF) [file ppat.1004890.s012.tif]
